# Supplementary material for: An immunohistochemical atlas of necroptotic pathway expression
Source: EMBO Mol Med. 2024 May 15;16(7):13. doi: 10.1038/s44321-024-00074-6 (PMC11250867; doi:10.1038/s44321-024-00074-6)
Supplement: Supplementary file 1 — Appendix [file 44321_2024_74_MOESM1_ESM.pdf]

Appendix material for "An immunohistochemical atlas of necroptotic pathway expression"

by Chiou & Al-Ani et al., 2024 *EMBO Molecular Medicine*

Appendix Figure S1……………………………………………………………………………Page 2

Appendix Figure S2……………………………………………………………………………Page 4

Appendix Figure S3……………………………………………………………………………Page 5

Appendix Figure S4…………………………………………………………………………...…Page 7

Appendix Figure S5……………………………………………………………………………Page 9

Appendix Table S1…………………………………………………………………………..Page 11

Appendix Table S2…………………………………………………………………………..Page 12

1. Mouse MLKL (clone 5A6)...…………...……………………………………………...Page 13
2. Mouse RIPK3 (clone 8G7)………...…………………………………………………..Page 17
3. Mouse RIPK3 (clone 1H12)……………...……………………………………………Page 21
4. Human and mouse RIPK1 (clone D94C12) + Sniper………………. …………………Page 25
5. Human and mouse RIPK1 (clone D94C12) + Sniper + MACH4………………………Page 29
6. Mouse Caspase-8 (clone 3B10) …………………………………...…………………Page 33
7. Mouse Caspase-8 (clone 1G12)………………………………………………………..Page 37
8. Human and mouse Caspase-8 (clone D53G2) ……………………………………….Page 55
9. Mouse RIPK3 8G7 + SMA (duo-IHC)…………………………………...………Page 59
10. Human MLKL (clone EPR17514) …………………………………………………....Page 78
11. Human MLKL (clone 10C2) ……………………………………………………..……Page 82
12. Human RIPK3 (clone E7A7F) …………………………………………………………..Page 86
13. Human Caspase-8 (clone B.925.8)……………………...……………………………..Page 90
14. Human cleaved Caspase-3 ……………………………………………………………Page 94
15. Ki67……………………………………………………………………………………...Page 98

Appendix Figure S1

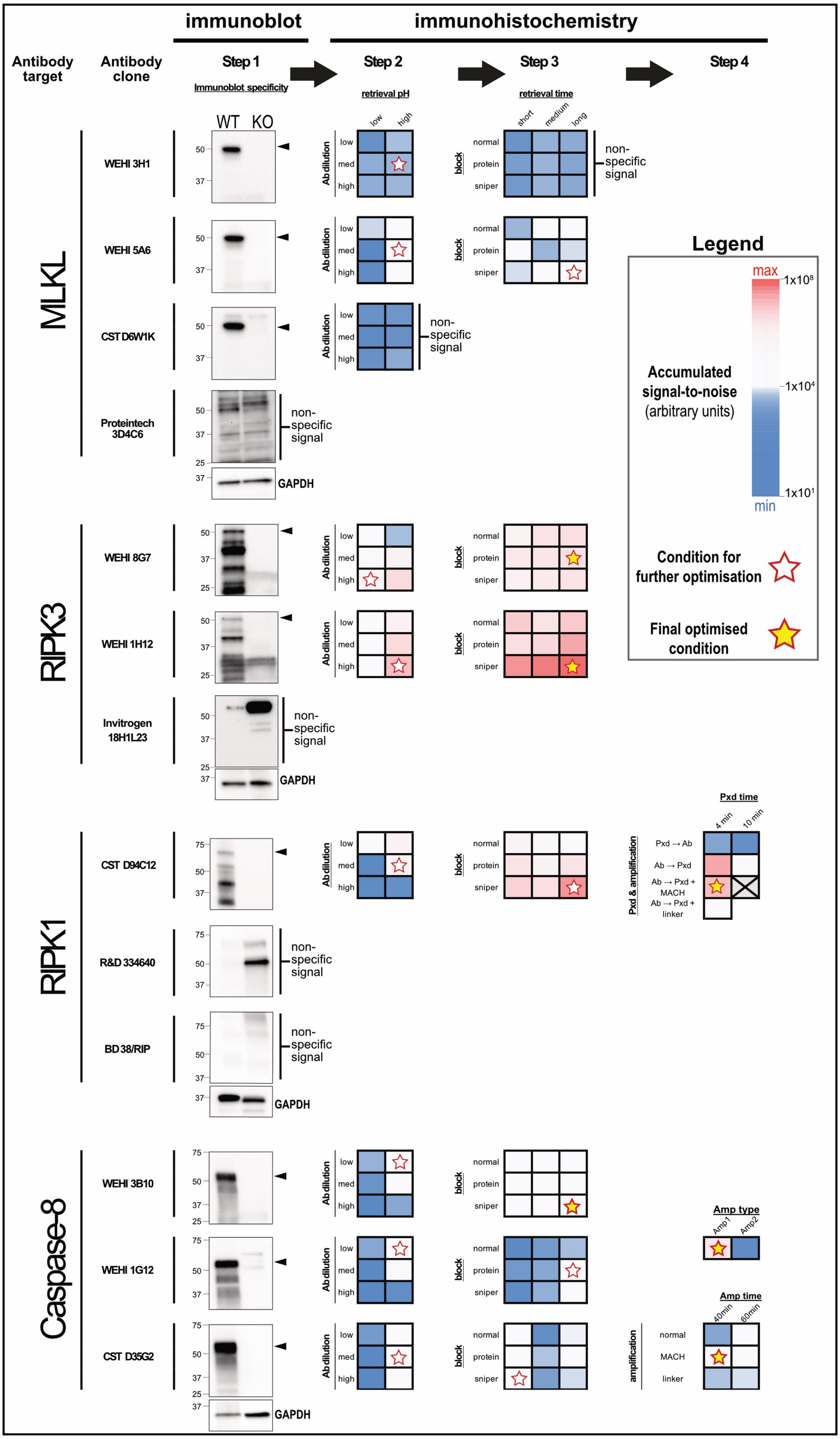

Appendix Figure S1. Optimisation pipeline for the immunohistochemical detection of the mouse necroptotic pathway. Summary of the steps used to test the specificity and to optimise the immunohistochemistry performance of monoclonal antibodies against mouse MLKL, RIPK3, RIPK1 and Caspase-8. Step 1: Immunoblot signals for each antibody on spleen homogenates from wild-type mice (WT) and *Mlkl*$^{-/-}$ or *Casp8*$^{-/-}$*Ripk3*$^{-/-}$ or *Casp8*$^{-/-}$*Ripk1*$^{-/-}$*Ripk3*$^{-/-}$ mice (KO). Arrowheads indicate the full-length protein of interest. Representative GAPDH immunoblots are shown as loading controls. Data are representative of n=1-2 immunoblots per antibody. Steps 2-4: Heatmaps depict the integrated signal-to-noise values derived from immunohistochemical signals for each antibody on WT versus KO spleen sections. Legend shows the heatmap color-to-value scale. Step 2 varied the primary antibody concentration and the antigen retrieval pH. Step 3 varied the blocking reagent and the antigen retrieval time. Step 4 varied the amplification technique, amplification time, peroxidase treatment time and whether peroxidase treatment preceded/superceded primary antibody incubation. For each antibody, the optimal condition at Step 2 (white stars) was the starting point for Step 3. Similarly, the optimal condition for Step 3 was the starting point for Step 4. Yellow stars indicate the final automated immunohistochemistry protocol stipulated in Appendix Table S2. Data in Steps 2-4 are representative of n=1-5 experiments per antibody per condition.

Appendix Figure S2

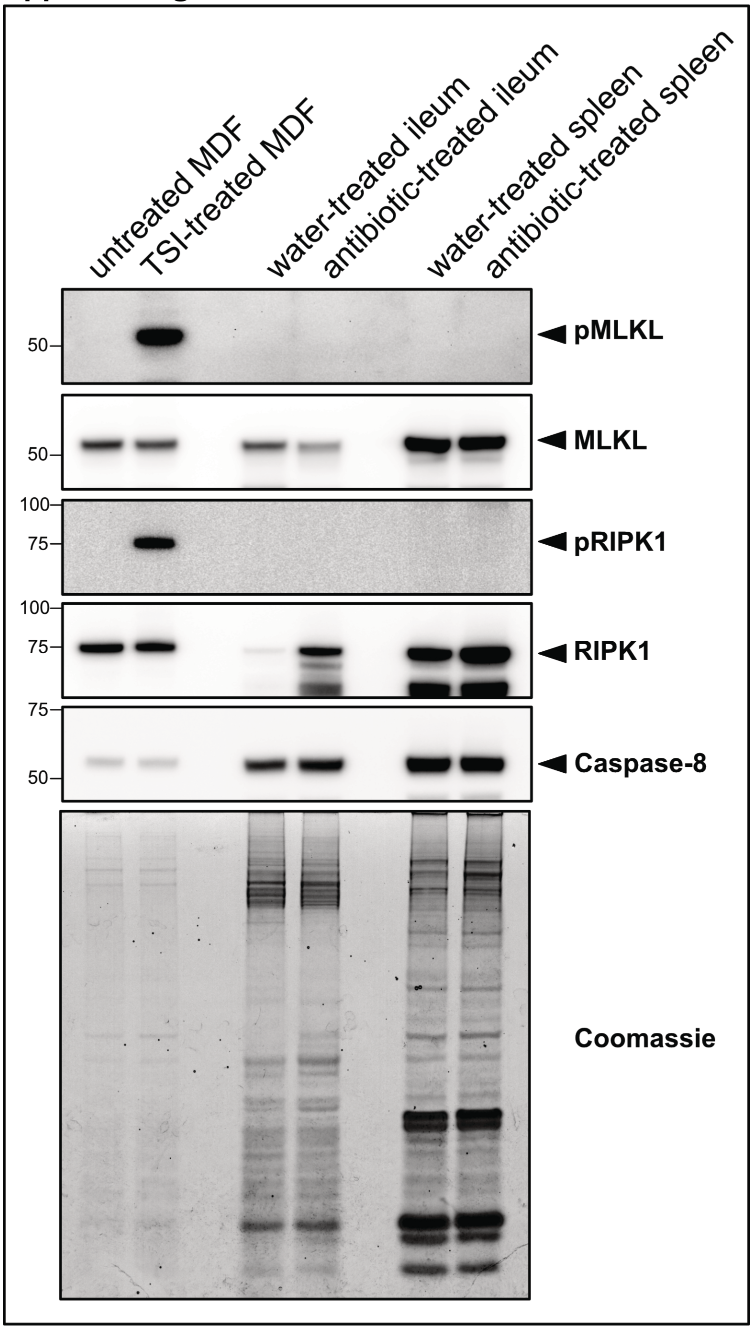

Appendix Figure S2. Antibiotic-induced clusters of epithelial RIPK1/3 are not necrosomes. Immunoblots for the indicated proteins in homogenates of the ileum and spleen of water- versus antibiotic-treated mice or in mouse dermal fibroblasts (MDF) undergoing TNF-induced necroptosis (TSI). Arrowheads indicate full-length proteins of interest. Coomassie staining of total protein content was used as a loading control. Data are representative of n=7 mice per tissue per group.

Appendix Figure S3

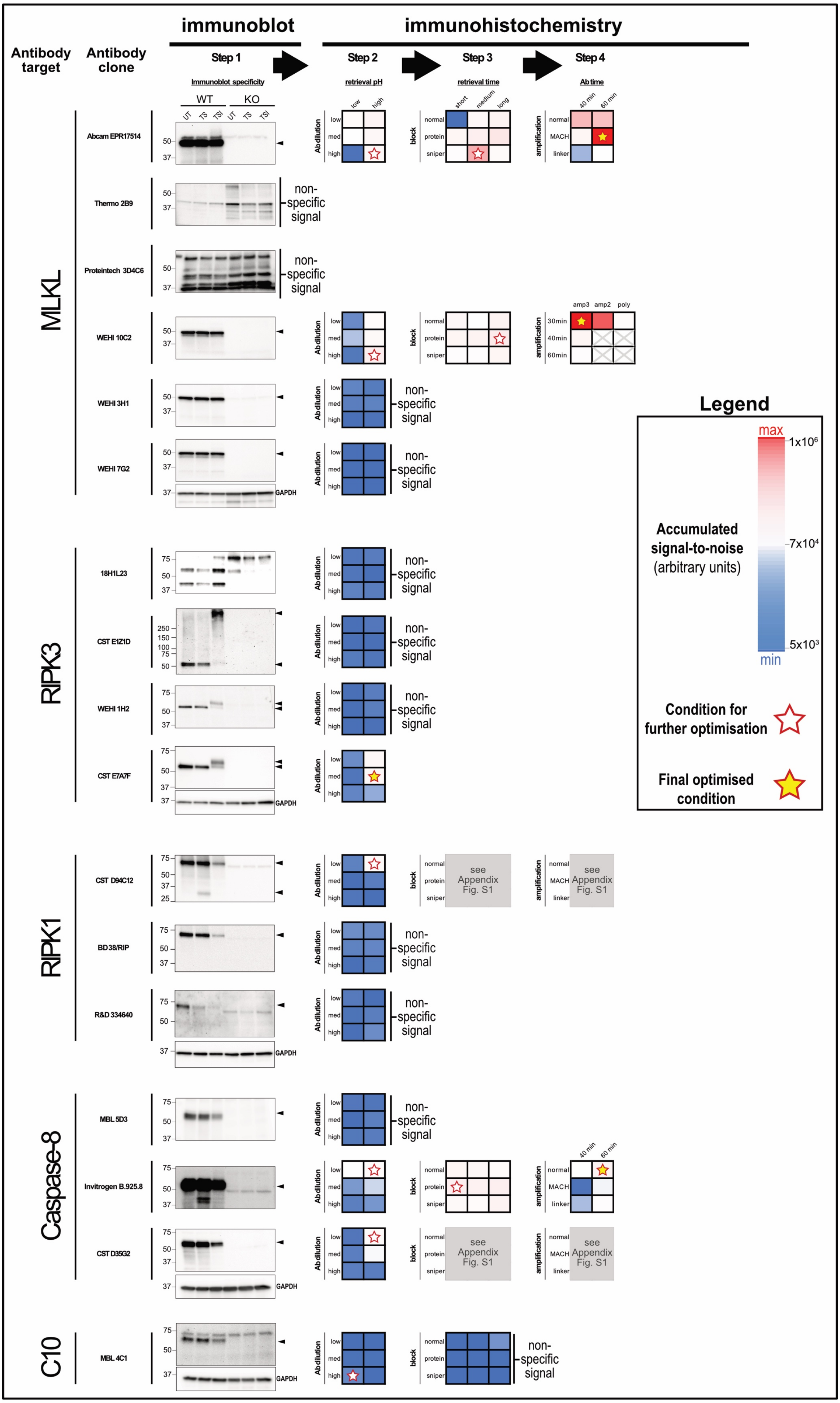

Appendix Figure S3. Standardised quality control and optimisation of the immunohistochemical detection of human necroptotic proteins. Summary of the optimisation for immunodetection with monoclonal antibodies against human MLKL, RIPK3, RIPK1, Caspase-8 and Caspase-10 (C10). Step 1: Immunoblot signals for each antibody on lysates from wild-type (WT) versus $MLKL^{-/-}$ or $RIPK1^{-/-}$ or $CASP8^{-/-}CASP10^{-/-}MLKL^{-/-}$ (KO) HT29 cells after treatment with the indicated stimuli. Arrowheads indicate the full-length protein of interest. Representative GAPDH immunoblots are shown as loading controls. Data are representative of n=1-2 immunoblots per antibody. Steps 2-4: Heatmaps depict the integrated signal-to-noise values derived from immunohistochemical signals for each antibody on WT versus KO HT29 cells. Legend shows the heatmap colour-to-value scale. Step 2 varied the primary antibody concentration and the antigen retrieval pH. Step 3 varied the blocking reagent and the antigen retrieval time. Step 4 varied the amplification technique, amplification time, peroxidase treatment time and whether peroxidase treatment preceded/superceded primary antibody incubation. For each antibody, the optimal condition at Step 2 (white stars) was the starting point for Step 3. Similarly, the optimal condition for Step 3 was the starting point for Step 4. Yellow stars indicate the final automated immunohistochemistry protocol stipulated in Appendix Table S2. Data in Steps 2-4 are representative of n=1-5 experiments per antibody per condition. The annotation 'see Appendix Figure S1' indicates optimisations that were only done using mouse tissues because these antibodies recognise both human and mouse orthologs.

Appendix Figure S4

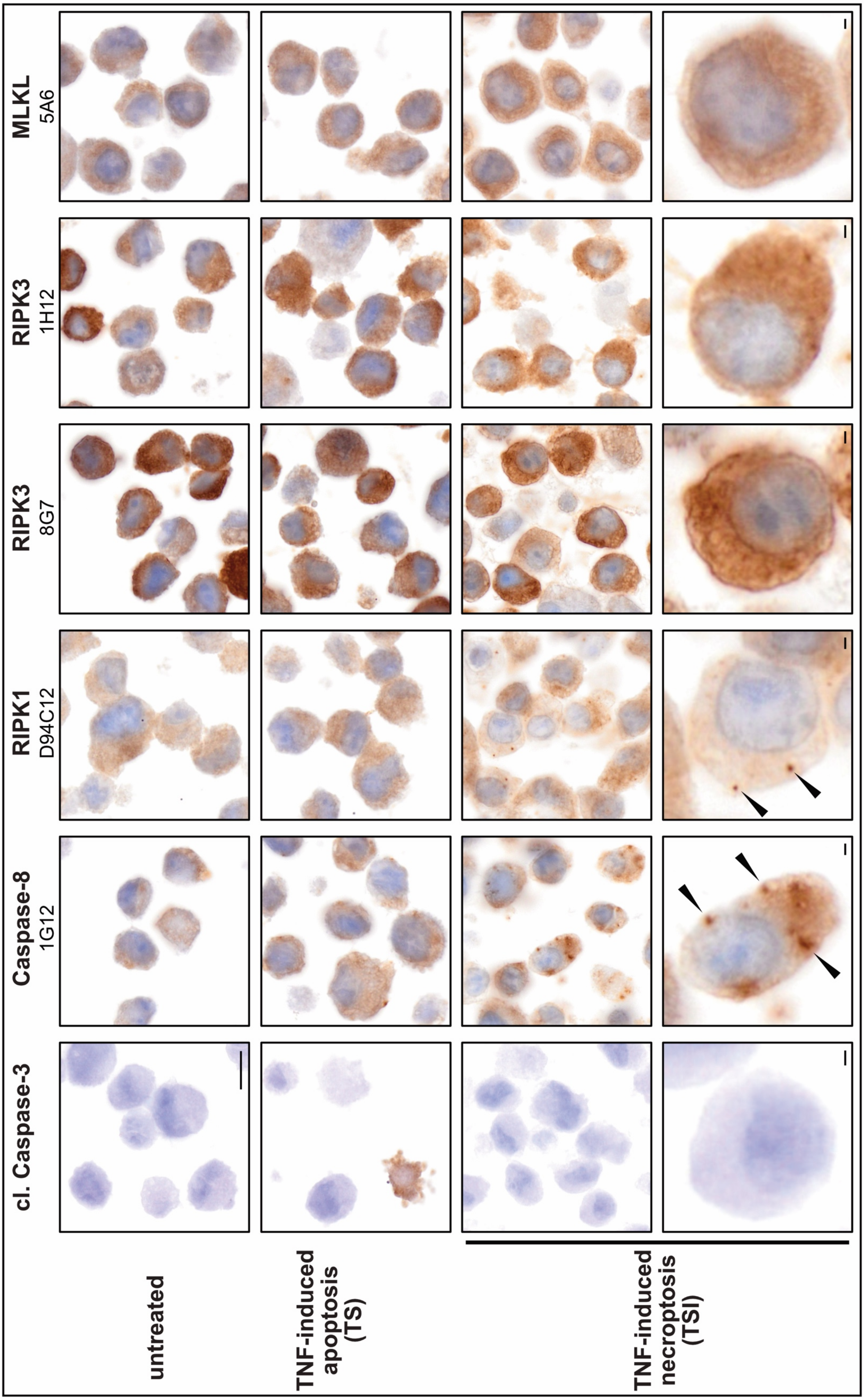

Appendix Figure S4. Automated immunohistochemistry to detect necroptotic signaling in mouse cells. Immunosignals of cleaved Caspase-3, Caspase-8, RIPK1, RIPK3 and MLKL in wild-type mouse dermal fibroblasts. Arrowheads indicate Caspase-8$^+$ or RIPK1$^+$ puncta that are presumed to be necrosomes. Data are representative of n=1 for each protein and treatment. Scale bars in lower magnification micrographs are 10μm. Scale bars in insets are 1μm.

Appendix Figure S5

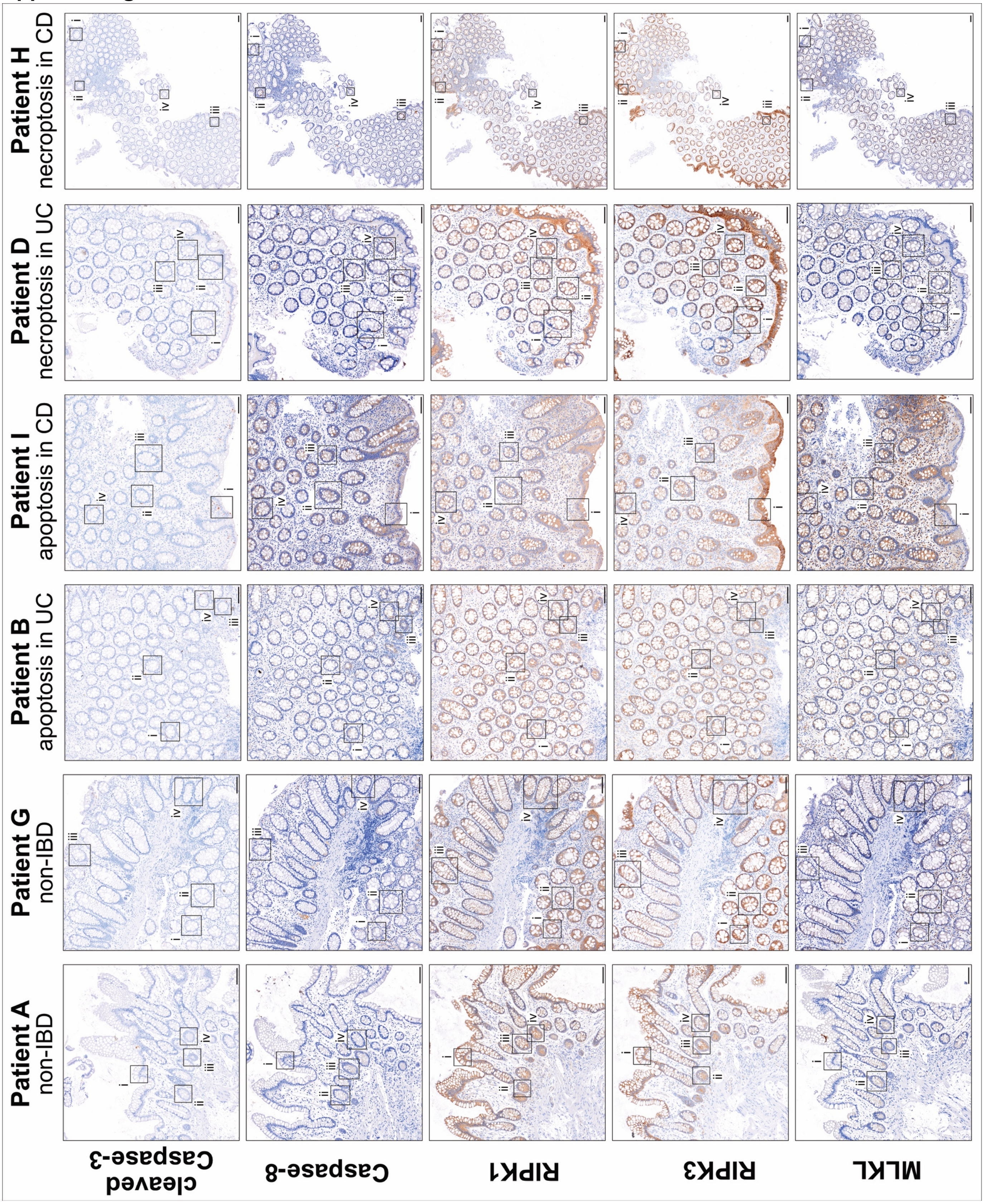

Appendix Figure S5. Atlas of the necroptotic pathway expression in a cohort of human intestinal biopsies. Immunohistochemistry for cleaved Caspase-3, Caspase-8 (clone B.925.8), RIPK1, RIPK3 and MLKL (clone EPR171514) on intestinal biopsies from the stipulated patients. The

insets show the location of each micrographs in Fig. **EV5** (annotated as i-v per biopsy). Scale bars are 100μm.

Appendix Table S1

LEGEND: - (not applicable), N.D. (not disclosed), IBD (inflammatory bowel disease), UC (ulcerative colitis), CD (Crohn's disease), HBI (Harvey-Bradshaw Index), SSCAI (Simple Clinical Colitis Activity Index), SES-CD (simple endoscopic score for Crohn's disease), Mayo (Mayo clinic score of disease activity for ulcerative colitis)

Study ID	Sex	Age (years)	IBD or non-IBD control	Scope indication	IBD classification	Clinical score	Endoscopic score	Biopsy endoscopic grading (and location)	Fecal calprotectin (µg/g) normal range 0-200	CRP (mg/L) normal range 0-5	Serum Albumin (g/L) normal range 34-54	IBD medications at time of endoscopy
A	Female	36	non-IBD control	Positive fecal occult blood test, rectal bleeding	-	-	-	non-inflamed (terminal ileum)	-	-	-	Nil
B	Male	71	IBD	IBD assessment	UC	SCCAI 9	Mayo 3	non-inflamed (descending colon), marginally inflamed (rectosigmoid), inflamed (rectum)	154	1.6	40	prednisolone 30 mg PO daily (weaning), budesonide 2 mg enemas, mesalazine suppository 1 g PO mane, mesalazine granules 1.5 g PO BD, infliximab 500 mg IV 8-weekly
C	Male	56	non-IBD control	Positive fecal occult blood test	-	-	-	non-inflamed (transverse colon)	-	-	-	Nil
D	Female	47	IBD	IBD assessment	UC	N.D.	Mayo 0	non-inflamed (sigmoid colon), marginally inflamed (proximal rectum), inflamed (distal rectum)	N.D.	N.D.	N.D.	Mesalazine 2.4 g PO daily, mesalazine 1 g PR nocte
E	Male	50	non-IBD control	Positive fecal occult blood test, rectal bleeding	-	-	-	non-inflamed (sigmoid colon), marginally inflamed (proximal rectum), inflamed (distal rectum)	-	-	-	Nil
F	Male	62	IBD	IBD assessment	CD	HBI 9	SES-CD 38	all inflamed due to disease severity- ileum, ascending colon, colon, sigmoid colon, rectum	926	41.5	32	ustekinumab 90 mg subcut 4-weekly, budesonide 9 mg PO daily
G	Male	31	non-IBD control	Iron deficiency	-	-	-	non-inflamed (rectum)	-	-	-	immunosuppressed for renal transplant: tacrolimus 1.5 mg PO mane, tacrolimus 1 mg PO daily, mycophenolate 500 mg PO BD, prednisolone 5 mg PO daily
H	Female	64	IBD	IBD assessment	CD	HBI 9	SES-CD 16	non-inflamed (rectum), marginally inflamed (rectum), inflamed (undilated rectal stricture)	63	3.8	39	Infliximab 400 mg IV 8-weekly, methotrexate 5 mg PO weekly
I	Female	30	IBD	IBD assessment	CD	HBI 3	SDS-CD 1	non-inflamed (rectum), marginally inflamed (transverse colon), inflamed (cecum)	323	14.4	39	Vedolizumab IV 4-weekly
J	Female	62	non-IBD control	Polyp surveillance	-	-	-	non-inflamed (ascending colon)	-	-	-	Nil

Appendix Table S1. Patient and clinical data related to **Fig.** 6.

Appendix Table S2. Autostainer immunohistochemistry protocols used in this manuscript.

Protocol - mMLKL 5A6

Version: 20/03/2023 3:37 PM

Dewax

Two phase dewax IHC

Solvent	Transport fluid	Temperature	Incubation top	Incubation bottom	Cycles
Clearify Clearing Agent	DI Water	25 °C	10 s	1 min	1

Two phase dewax wash IHC

Reagent	Incubation	Cycles
DI Water	5 s	1

Target retrieval

Target retrieval IHC

Reagent	Temperature	Incubation	Cooling fluid
EnV FLEX TRS, High pH	97 °C	40 min	DI Water

Non target retrieval wash IHC

Staining

Wash

Reagent	Incubation	Cycles
Wash Buffer	2:40 min	2

Enzyme pre-treatment

Reagent	Incubation
Background Sniper BS966L (Biocare Medical) working	10 min

Wash

Reagent	Incubation	Cycles
Wash Buffer	2 min	10

Wash

Endogenous enzyme block

Wash

Protein block

Wash

Protocol details

Agilent Dako

Primary antibody

Reagent	Incubation
mMLKL 5A6 1:200 working	1 h

Wash

Reagent	Incubation	Cycles
Wash Buffer	2 min	10

Wash

Wash

Wash

Primary antibody

Reagent	Incubation
Flex Peroxidase Blcok working	4 min

Wash

Reagent	Incubation	Cycles
Wash Buffer	2 min	10

Wash

Wash

Wash

Primary antibody

Reagent	Incubation
Protein Block X0909 (Dako) RTU	10 min

Wash

Reagent	Incubation	Cycles
Wash Buffer	2 min	10

Wash

Wash

Wash

Endogenous enzyme block

Wash

Secondary reagent

Wash

Wash

Wash

Wash

Secondary reagent

Wash

Wash

Wash

Wash

Labeled polymer

Reagent	Incubation
HRP (anti-rat)	30 min

Wash

Reagent	Incubation	Cycles
Wash Buffer	2 min	10

Wash

Reagent	Incubation	Cycles
Wash Buffer	2 min	10

Wash

Reagent	Incubation	Cycles
DI Water	31 s	1

Wash

Reagent	Incubation	Cycles
Wash Buffer	2 min	10

Wash

Wash

Wash

Substrate chromogen

Reagent	Incubation
EnV FLEX Substrate Working Solution	10 min

Wash

Substrate chromogen

Wash

Reagent	Incubation	Cycles
Wash Buffer	2 min	10

Wash

Reagent	Incubation	Cycles
DI Water	31 s	1

Wash

Reagent	Incubation	Cycles
Wash Buffer	2 min	10

Counterstaining

Protocol - anti-mouse RIPK3 (WEHI 8G7)

Version: 27/02/2023 1:17 PM

Dewax

Two phase dewax IHC

Solvent	Transport fluid	Temperature	Incubation top	Incubation bottom	Cycles
Clearify Clearing Agent	DI Water	25 °C	10 s	1 min	1

Two phase dewax wash IHC

Reagent	Incubation	Cycles
DI Water	5 s	1

Target retrieval

Target retrieval IHC

Reagent	Temperature	Incubation	Cooling fluid
EnVision FLEX TRS, Low pH	97 °C	40 min	DI Water

Non target retrieval wash IHC

Staining

Wash

Reagent	Incubation	Cycles
Wash Buffer	2:40 min	2

Enzyme pre-treatment

Reagent	Incubation
Protein Block X0909 (Dako) RTU working	10 min

Wash

Reagent	Incubation	Cycles
Wash Buffer	2 min	10

Wash

Endogenous enzyme block

Wash

Protein block

Wash

Primary antibody

Protocol details

Reagent	Incubation
mRIPK3 8G7 working 1:500	1 h

Wash

Reagent	Incubation	Cycles
Wash Buffer	2 min	10

Wash

Wash

Wash

Primary antibody

Reagent	Incubation
Flex Peroxidase Blcok working	4 min

Wash

Reagent	Incubation	Cycles
Wash Buffer	2 min	10

Wash

Wash

Wash

Primary antibody

Reagent	Incubation
Protein Block X0909 (Dako) RTU	10 min

Wash

Reagent	Incubation	Cycles
Wash Buffer	2 min	10

Wash

Wash

Wash

Endogenous enzyme block

Wash

Secondary reagent

Wash

Wash

Wash

Wash

Secondary reagent

Wash

Wash

Wash

Wash

Labeled polymer

Reagent	Incubation
HRP (anti-rat)	30 min

Wash

Reagent	Incubation	Cycles
Wash Buffer	2 min	10

Wash

Reagent	Incubation	Cycles
Wash Buffer	2 min	10

Wash

Reagent	Incubation	Cycles
DI Water	31 s	1

Wash

Reagent	Incubation	Cycles
Wash Buffer	2 min	10

Wash

Wash

Wash

Substrate chromogen

Reagent	Incubation
EnV FLEX Substrate Working Solution	10 min

Wash

Substrate chromogen

Wash

Reagent	Incubation	Cycles

Wash Buffer	2 min	10

Wash

Reagent	Incubation	Cycles
DI Water	31 s	1

Wash

Reagent	Incubation	Cycles
Wash Buffer	2 min	10

Counterstaining

Protocol - mRIPK3 1H12

Version: 20/03/2023 3:50 PM

Dewax

Two phase dewax IHC

Solvent	Transport fluid	Temperature	Incubation top	Incubation bottom	Cycles
Clearify Clearing Agent	DI Water	25 °C	10 s	1 min	1

Two phase dewax wash IHC

Reagent	Incubation	Cycles
DI Water	5 s	1

Target retrieval

Target retrieval IHC

Reagent	Temperature	Incubation	Cooling fluid
EnV FLEX TRS, High pH	97 °C	30 min	DI Water

Non target retrieval wash IHC

Staining

Wash

Reagent	Incubation	Cycles
Wash Buffer	2:40 min	2

Enzyme pre-treatment

Reagent	Incubation
Background Sniper BS966L (Biocare Medical) working	10 min

Wash

Reagent	Incubation	Cycles
Wash Buffer	2 min	10

Wash

Endogenous enzyme block

Wash

Protein block

Wash

Protocol details

Agilent Dako

Primary antibody

Reagent	Incubation
mRIPK3 1H12 1:100 working	1 h

Wash

Reagent	Incubation	Cycles
Wash Buffer	2 min	10

Wash

Wash

Wash

Primary antibody

Reagent	Incubation
Flex Peroxidase Blcok working	4 min

Wash

Reagent	Incubation	Cycles
Wash Buffer	2 min	10

Wash

Wash

Wash

Primary antibody

Reagent	Incubation
Protein Block X0909 (Dako) RTU	10 min

Wash

Reagent	Incubation	Cycles
Wash Buffer	2 min	10

Wash

Wash

Wash

Endogenous enzyme block

Wash

Secondary reagent

Wash

Protocol details

Wash

Wash

Wash

Secondary reagent

Wash

Wash

Wash

Wash

Labeled polymer

Reagent	Incubation
HRP (anti-rat)	30 min

Wash

Reagent	Incubation	Cycles
Wash Buffer	2 min	10

Wash

Reagent	Incubation	Cycles
Wash Buffer	2 min	10

Wash

Reagent	Incubation	Cycles
DI Water	31 s	1

Wash

Reagent	Incubation	Cycles
Wash Buffer	2 min	10

Wash

Wash

Wash

Substrate chromogen

Reagent	Incubation
EnV FLEX Substrate Working Solution	10 min

Wash

Substrate chromogen

Wash

Reagent	Incubation	Cycles
Wash Buffer	2 min	10

Wash

Reagent	Incubation	Cycles
DI Water	31 s	1

Wash

Reagent	Incubation	Cycles
Wash Buffer	2 min	10

Counterstaining

Protocol - RIPK1 D94C12-Sniper

Version: 7/12/2022 11:29 AM

Dewax

Two phase dewax IHC

Solvent	Transport fluid	Temperature	Incubation top	Incubation bottom	Cycles
Clearify Clearing Agent	DI Water	25 °C	10 s	1 min	1

Two phase dewax wash IHC

Reagent	Incubation	Cycles
DI Water	5 s	1

Target retrieval

Target retrieval IHC

Reagent	Temperature	Incubation	Cooling fluid
EnV FLEX TRS, High pH	97 °C	40 min	DI Water

Non target retrieval wash IHC

Staining

Wash

Reagent	Incubation	Cycles
Wash Buffer	2:40 min	2

Enzyme pre-treatment

Reagent	Incubation
Background Sniper BS966L (Biocare Medical) working	10 min

Wash

Reagent	Incubation	Cycles
Wash Buffer	2 min	10

Wash

Endogenous enzyme block

Wash

Protein block

Wash

Protocol details

Primary antibody

Reagent	Incubation
RIPK1 D94C12 working 1:200	1 h

Wash

Reagent	Incubation	Cycles
Wash Buffer	2 min	10

Wash

Wash

Wash

Primary antibody

Reagent	Incubation
Flex Peroxidase Blcok working	4 min

Wash

Reagent	Incubation	Cycles
Wash Buffer	2 min	10

Wash

Wash

Wash

Primary antibody

Reagent	Incubation
Rabbit Labelled Polymer - HRP (Dako K4003)	30 min

Wash

Reagent	Incubation	Cycles
Wash Buffer	2 min	10

Wash

Wash

Wash

Endogenous enzyme block

Wash

Secondary reagent

Wash

Wash

Wash

Wash

Secondary reagent

Wash

Wash

Wash

Wash

Labeled polymer

Reagent	Incubation
Buffer Only	3 min

Wash

Reagent	Incubation	Cycles
Wash Buffer	2 min	10

Wash

Reagent	Incubation	Cycles
Wash Buffer	2 min	10

Wash

Reagent	Incubation	Cycles
DI Water	31 s	1

Wash

Reagent	Incubation	Cycles
Wash Buffer	2 min	10

Wash

Wash

Wash

Substrate chromogen

Reagent	Incubation
EnV FLEX Substrate Working Solution	10 min

Wash

Substrate chromogen

Wash

Reagent	Incubation	Cycles
Wash Buffer	2 min	10

Wash

Reagent	Incubation	Cycles
DI Water	31 s	1

Wash

Reagent	Incubation	Cycles
Wash Buffer	2 min	10

Counterstaining

Protocol - RIPK1 D94C12-MACH Sniper

Version: 8/03/2024 3:33 PM

Dewax

Two phase dewax IHC

Solvent	Transport fluid	Temperature	Incubation top	Incubation bottom	Cycles
Clearify Clearing Agent	DI Water	25 °C	10 s	1 min	1

Two phase dewax wash IHC

Reagent	Incubation	Cycles
DI Water	5 s	1

Target retrieval

Target retrieval IHC

Reagent	Temperature	Incubation	Cooling fluid
EnV FLEX TRS, High pH	97 °C	40 min	DI Water

Non target retrieval wash IHC

Staining

Wash

Reagent	Incubation	Cycles
Wash Buffer	2:40 min	2

Enzyme pre-treatment

Reagent	Incubation
Background Sniper BS966L (Biocare Medical) working	10 min

Wash

Reagent	Incubation	Cycles
Wash Buffer	2 min	10

Wash

Endogenous enzyme block

Wash

Protein block

Wash

Protocol details

Primary antibody

Reagent	Incubation
RIPK1 D94C12 working 1:200	1 h

Wash

Reagent	Incubation	Cycles
Wash Buffer	2 min	10

Wash

Wash

Wash

Primary antibody

Reagent	Incubation
Flex Peroxidase Blcok working	4 min

Wash

Reagent	Incubation	Cycles
Wash Buffer	2 min	10

Wash

Wash

Wash

Primary antibody

Reagent	Incubation
MACH4 Rabbit RTU	30 min

Wash

Reagent	Incubation	Cycles
Wash Buffer	2 min	10

Wash

Wash

Wash

Endogenous enzyme block

Wash

Secondary reagent

Wash

Wash

Wash

Wash

Secondary reagent

Wash

Wash

Wash

Wash

Labeled polymer

Reagent	Incubation
Buffer Only	3 min

Wash

Reagent	Incubation	Cycles
Wash Buffer	2 min	10

Wash

Reagent	Incubation	Cycles
Wash Buffer	2 min	10

Wash

Reagent	Incubation	Cycles
DI Water	31 s	1

Wash

Reagent	Incubation	Cycles
Wash Buffer	2 min	10

Wash

Wash

Wash

Substrate chromogen

Reagent	Incubation
Washing Buffer only	10 min

Wash

Substrate chromogen

Wash

Reagent	Incubation	Cycles
Wash Buffer	2 min	10

Wash

Reagent	Incubation	Cycles
DI Water	31 s	1

Wash

Reagent	Incubation	Cycles
Wash Buffer	2 min	10

Counterstaining

Walter & Eliza Hall Institute of Medical Research
Royal Parade
PARKVILLE VIC 3052

Protocol - mCasp8 3B10

Version: 24/03/2023 4:07 PM

Dewax

Two phase dewax IHC

Solvent	Transport fluid	Temperature	Incubation top	Incubation bottom	Cycles
Clearify Clearing Agent	DI Water	25 °C	10 s	1 min	1

Two phase dewax wash IHC

Reagent	Incubation	Cycles
DI Water	5 s	1

Target retrieval

Target retrieval IHC

Reagent	Temperature	Incubation	Cooling fluid
EnV FLEX TRS, High pH	97 °C	40 min	DI Water

Non target retrieval wash IHC

Staining

Wash

Reagent	Incubation	Cycles
Wash Buffer	2:40 min	2

Enzyme pre-treatment

Reagent	Incubation
Background Sniper BS966L (Biocare Medical) working	10 min

Wash

Reagent	Incubation	Cycles
Wash Buffer	2 min	10

Wash

Endogenous enzyme block

Wash

Protein block

Wash

Protocol details

Agilent Dako

Primary antibody

Reagent	Incubation
mCasp8 3B10 1:200 Working	1 h

Wash

Reagent	Incubation	Cycles
Wash Buffer	2 min	10

Wash

Wash

Wash

Primary antibody

Reagent	Incubation
Flex Peroxidase Blcok working	4 min

Wash

Reagent	Incubation	Cycles
Wash Buffer	2 min	10

Wash

Wash

Wash

Primary antibody

Reagent	Incubation
Protein Block X0909 (Dako) RTU	10 min

Wash

Reagent	Incubation	Cycles
Wash Buffer	2 min	10

Wash

Wash

Wash

Endogenous enzyme block

Wash

Secondary reagent

Wash

Wash

Wash

Wash

Secondary reagent

Wash

Wash

Wash

Wash

Labeled polymer

Reagent	Incubation
HRP (anti-rat)	30 min

Wash

Reagent	Incubation	Cycles
Wash Buffer	2 min	10

Wash

Reagent	Incubation	Cycles
Wash Buffer	2 min	10

Wash

Reagent	Incubation	Cycles
DI Water	31 s	1

Wash

Reagent	Incubation	Cycles
Wash Buffer	2 min	10

Wash

Wash

Wash

Substrate chromogen

Reagent	Incubation
EnV FLEX Substrate Working Solution	10 min

Wash

Substrate chromogen

Wash

Protocol details

Agilent Dako

Reagent	Incubation	Cycles
Wash Buffer	2 min	10

Wash

Reagent	Incubation	Cycles
DI Water	31 s	1

Wash

Reagent	Incubation	Cycles
Wash Buffer	2 min	10

Counterstaining

Protocol - mCasp8_1G12

Version: 2/06/2023 12:50 PM

Dewax

Two phase dewax IHC

Solvent	Transport fluid	Temperature	Incubation top	Incubation bottom	Cycles
Clearify Clearing Agent	DI Water	25 °C	10 s	1 min	1

Two phase dewax wash IHC

Reagent	Incubation	Cycles
DI Water	5 s	1

Target retrieval

Target retrieval IHC

Reagent	Temperature	Incubation	Cooling fluid
EnV FLEX TRS, High pH	97 °C	40 min	DI Water

Non target retrieval wash IHC

Staining

Wash

Reagent	Incubation	Cycles
Wash Buffer	2:40 min	2

Enzyme pre-treatment

Wash

Endogenous enzyme block

Wash

Protein block

Reagent	Incubation
Protein Block RTU X0909 (Dako)	10 min

Wash

Reagent	Incubation	Cycles
Wash Buffer	2 min	10

Primary antibody

Reagent	Incubation

mCasp8_1G12 1:200	1 h	
**Wash**		
**Reagent**	**Incubation**	**Cycles**
Wash Buffer	2 min	10
**Wash**		
**Wash**		
**Wash**		
**Primary antibody**		
**Reagent**	**Incubation**	
Flex Peroxidase Blcok working	4 min	
**Wash**		
**Reagent**	**Incubation**	**Cycles**
Wash Buffer	2 min	10
**Wash**		
**Wash**		
**Wash**		
**Primary antibody**		
**Reagent**	**Incubation**	
Protein Block X0909 (Dako) RTU	10 min	
**Wash**		
**Reagent**	**Incubation**	**Cycles**
Wash Buffer	2 min	10
**Wash**		
**Wash**		
**Wash**		
**Endogenous enzyme block**		
**Wash**		
**Wash**		
**Wash**		
**Wash**		
**Secondary reagent**		

Wash

Wash

Wash

Wash

Secondary reagent

Wash

Wash

Wash

Wash

Labeled polymer

Reagent	Incubation
HRP (anti-rat)	30 min

Wash

Reagent	Incubation	Cycles
Wash Buffer	2 min	10

Wash

Reagent	Incubation	Cycles
Wash Buffer	2 min	10

Wash

Reagent	Incubation	Cycles
DI Water	31 s	1

Wash

Reagent	Incubation	Cycles
Wash Buffer	2 min	10

Substrate chromogen

Reagent	Incubation
Opal 540 working 1:500	10 min

Wash

Reagent	Incubation	Cycles
Wash Buffer	2 min	10

Substrate chromogen

Wash

Wash

Reagent	Incubation	Cycles
DI Water	31 s	1

Wash

Reagent	Incubation	Cycles
Wash Buffer	2 min	10

Endogenous enzyme block

Wash

Wash

Wash

Wash

Protein block

Wash

Primary antibody

Reagent	Incubation
DIG HRP 1:500 WB	30 min

Wash

Reagent	Incubation	Cycles
Wash Buffer	2 min	10

Wash

Wash

Wash

Primary antibody

Wash

Wash

Wash

Wash

Primary antibody

Wash

Wash

Wash

Wash

Endogenous enzyme block

Wash

Wash

Wash

Wash

Secondary reagent

Wash

Wash

Wash

Wash

Secondary reagent

Wash

Wash

Wash

Wash

Labeled polymer

Reagent	Incubation
Buffer Only	3 min

Wash

Reagent	Incubation	Cycles
Wash Buffer	2 min	10

Wash

Reagent	Incubation	Cycles
Wash Buffer	2 min	10

Wash

Reagent	Incubation	Cycles
DI Water	31 s	1

Wash

Reagent	Incubation	Cycles
Wash Buffer	2 min	10

Substrate chromogen

Reagent	Incubation
EnV FLEX Substrate	

Working Solution	5 min

Wash

Reagent	Incubation	Cycles
Wash Buffer	2 min	10

Substrate chromogen

Wash

Wash

Reagent	Incubation	Cycles
DI Water	31 s	1

Wash

Reagent	Incubation	Cycles
Wash Buffer	2 min	10

Endogenous enzyme block

Wash

Wash

Wash

Wash

Protein block

Wash

Primary antibody

Wash

Wash

Wash

Wash

Primary antibody

Wash

Wash

Wash

Wash

Primary antibody

Wash

Wash

**Wash**
**Wash**
**Endogenous enzyme block**
**Wash**
**Wash**
**Wash**
**Wash**
**Secondary reagent**
**Wash**
**Wash**
**Wash**
**Wash**
**Secondary reagent**
**Wash**
**Wash**
**Wash**
**Wash**
**Labeled polymer**
**Wash**
**Wash**
**Wash**
**Wash**
**Substrate chromogen**
**Wash**
**Substrate chromogen**
**Wash**
**Wash**
**Wash**
**Endogenous enzyme block**
**Wash**
**Wash**
**Wash**

Protocol details

**Wash**
**Protein block**
**Wash**
**Primary antibody**
**Wash**
**Wash**
**Wash**
**Wash**
**Primary antibody**
**Wash**
**Wash**
**Wash**
**Wash**
**Primary antibody**
**Wash**
**Wash**
**Wash**
**Wash**
**Endogenous enzyme block**
**Wash**
**Wash**
**Wash**
**Wash**
**Secondary reagent**
**Wash**
**Wash**
**Wash**
**Wash**
**Secondary reagent**
**Wash**
**Wash**
**Wash**

**Wash**
**Labeled polymer**
**Wash**
**Wash**
**Wash**
**Wash**
**Substrate chromogen**
**Wash**
**Substrate chromogen**
**Wash**
**Wash**
**Wash**
**Endogenous enzyme block**
**Wash**
**Wash**
**Wash**
**Wash**
**Protein block**
**Wash**
**Primary antibody**
**Wash**
**Wash**
**Wash**
**Wash**
**Primary antibody**
**Wash**
**Wash**
**Wash**
**Wash**
**Primary antibody**
**Wash**
**Wash**

**Wash**
**Wash**
**Endogenous enzyme block**
**Wash**
**Wash**
**Wash**
**Wash**
**Secondary reagent**
**Wash**
**Wash**
**Wash**
**Wash**
**Secondary reagent**
**Wash**
**Wash**
**Wash**
**Wash**
**Labeled polymer**
**Wash**
**Wash**
**Wash**
**Wash**
**Substrate chromogen**
**Wash**
**Substrate chromogen**
**Wash**
**Wash**
**Wash**
**Endogenous enzyme block**
**Wash**
**Wash**
**Wash**

Protocol details

**Wash**
**Protein block**
**Wash**
**Primary antibody**
**Wash**
**Wash**
**Wash**
**Wash**
**Primary antibody**
**Wash**
**Wash**
**Wash**
**Wash**
**Primary antibody**
**Wash**
**Wash**
**Wash**
**Wash**
**Endogenous enzyme block**
**Wash**
**Wash**
**Wash**
**Wash**
**Secondary reagent**
**Wash**
**Wash**
**Wash**
**Wash**
**Secondary reagent**
**Wash**
**Wash**
**Wash**

**Wash**
**Labeled polymer**
**Wash**
**Wash**
**Wash**
**Wash**
**Substrate chromogen**
**Wash**
**Substrate chromogen**
**Wash**
**Wash**
**Wash**
**Endogenous enzyme block**
**Wash**
**Wash**
**Wash**
**Wash**
**Protein block**
**Wash**
**Primary antibody**
**Wash**
**Wash**
**Wash**
**Wash**
**Primary antibody**
**Wash**
**Wash**
**Wash**
**Wash**
**Primary antibody**
**Wash**
**Wash**

**Wash**
**Wash**
**Endogenous enzyme block**
**Wash**
**Wash**
**Wash**
**Wash**
**Secondary reagent**
**Wash**
**Wash**
**Wash**
**Wash**
**Secondary reagent**
**Wash**
**Wash**
**Wash**
**Wash**
**Labeled polymer**
**Wash**
**Wash**
**Wash**
**Wash**
**Substrate chromogen**
**Wash**
**Substrate chromogen**
**Wash**
**Wash**
**Wash**
**Endogenous enzyme block**
**Wash**
**Wash**
**Wash**

Walter & Eliza Hall Institute of Medical Research
Royal Parade
PARKVILLE VIC 3052

**Wash**
**Protein block**
**Wash**
**Primary antibody**
**Wash**
**Wash**
**Wash**
**Wash**
**Primary antibody**
**Wash**
**Wash**
**Wash**
**Wash**
**Primary antibody**
**Wash**
**Wash**
**Wash**
**Wash**
**Endogenous enzyme block**
**Wash**
**Wash**
**Wash**
**Wash**
**Secondary reagent**
**Wash**
**Wash**
**Wash**
**Wash**
**Secondary reagent**
**Wash**
**Wash**
**Wash**

Protocol details

**Wash**
**Labeled polymer**
**Wash**
**Wash**
**Wash**
**Wash**
**Substrate chromogen**
**Wash**
**Substrate chromogen**
**Wash**
**Wash**
**Wash**
**Endogenous enzyme block**
**Wash**
**Wash**
**Wash**
**Wash**
**Protein block**
**Wash**
**Primary antibody**
**Wash**
**Wash**
**Wash**
**Wash**
**Primary antibody**
**Wash**
**Wash**
**Wash**
**Wash**
**Primary antibody**
**Wash**
**Wash**

**Wash**
**Wash**
**Endogenous enzyme block**
**Wash**
**Wash**
**Wash**
**Wash**
**Secondary reagent**
**Wash**
**Wash**
**Wash**
**Wash**
**Secondary reagent**
**Wash**
**Wash**
**Wash**
**Wash**
**Labeled polymer**
**Wash**
**Wash**
**Wash**
**Wash**
**Substrate chromogen**
**Wash**
**Substrate chromogen**
**Wash**
**Wash**
**Wash**
**Endogenous enzyme block**
**Wash**
**Wash**
**Wash**

Protocol details

**Wash**
**Protein block**
**Wash**
**Primary antibody**
**Wash**
**Wash**
**Wash**
**Wash**
**Primary antibody**
**Wash**
**Wash**
**Wash**
**Wash**
**Primary antibody**
**Wash**
**Wash**
**Wash**
**Wash**
**Endogenous enzyme block**
**Wash**
**Wash**
**Wash**
**Wash**
**Secondary reagent**
**Wash**
**Wash**
**Wash**
**Wash**
**Secondary reagent**
**Wash**
**Wash**
**Wash**

**Wash**
**Labeled polymer**
**Wash**
**Wash**
**Wash**
**Wash**
**Substrate chromogen**
**Wash**
**Substrate chromogen**
**Wash**
**Wash**
**Wash**

Counterstaining

Protocol - h_mCaspase8 (CST D35G2)

Version: 24/02/2023 3:22 PM

Dewax

Two phase dewax IHC

Solvent	Transport fluid	Temperature	Incubation top	Incubation bottom	Cycles
Clearify Clearing Agent	DI Water	25 °C	10 s	1 min	1

Two phase dewax wash IHC

Reagent	Incubation	Cycles
DI Water	5 s	1

Target retrieval

Target retrieval IHC

Reagent	Temperature	Incubation	Cooling fluid
EnV FLEX TRS, High pH	97 °C	20 min	DI Water

Non target retrieval wash IHC

Staining

Wash

Reagent	Incubation	Cycles
Wash Buffer	2:40 min	2

Enzyme pre-treatment

Reagent	Incubation
Background Sniper BS966L (Biocare Medical) working	10 min

Wash

Reagent	Incubation	Cycles
Wash Buffer	2 min	10

Wash

Endogenous enzyme block

Wash

Protein block

Wash

Protocol details

Primary antibody

Reagent	Incubation
h_mCaspase8 (CST D35G2) working 1:200	1 h

Wash

Reagent	Incubation	Cycles
Wash Buffer	2 min	10

Wash

Wash

Wash

Primary antibody

Reagent	Incubation
Flex Peroxidase Blcok working	4 min

Wash

Reagent	Incubation	Cycles
Wash Buffer	2 min	10

Wash

Wash

Wash

Primary antibody

Reagent	Incubation
MACH4 Rabbit RTU	30 min

Wash

Reagent	Incubation	Cycles
Wash Buffer	2 min	10

Wash

Wash

Wash

Endogenous enzyme block

Wash

Secondary reagent

Wash

Wash

Wash

Wash

Secondary reagent

Wash

Wash

Wash

Wash

Labeled polymer

Reagent	Incubation
Buffer Only	3 min

Wash

Reagent	Incubation	Cycles
Wash Buffer	2 min	10

Wash

Reagent	Incubation	Cycles
Wash Buffer	2 min	10

Wash

Reagent	Incubation	Cycles
DI Water	31 s	1

Wash

Reagent	Incubation	Cycles
Wash Buffer	2 min	10

Wash

Wash

Wash

Substrate chromogen

Reagent	Incubation
EnV FLEX Substrate Working Solution	10 min

Wash

Substrate chromogen

Wash

Reagent	Incubation	Cycles
Wash Buffer	2 min	10

Wash

Reagent	Incubation	Cycles
DI Water	31 s	1

Wash

Reagent	Incubation	Cycles
Wash Buffer	2 min	10

Counterstaining

Protocol - mRIPK3 8G7+ SMA

Version: 27/06/2023 3:34 PM

Dewax

Two phase dewax IHC

Solvent	Transport fluid	Temperature	Incubation top	Incubation bottom	Cycles
Clearify Clearing Agent	DI Water	25 °C	10 s	1 min	1

Two phase dewax wash IHC

Reagent	Incubation	Cycles
DI Water	5 s	1

Target retrieval

Target retrieval IHC

Reagent	Temperature	Incubation	Cooling fluid
EnVision FLEX TRS, Low pH	97 °C	40 min	DI Water

Non target retrieval wash IHC

Staining

Wash

Reagent	Incubation	Cycles
Wash Buffer	2:40 min	2

Enzyme pre-treatment

Reagent	Incubation
Protein Block X0909 (Dako) RTU working	10 min

Wash

Reagent	Incubation	Cycles
Wash Buffer	2 min	10

Endogenous enzyme block

Wash

Protein block

Wash

Primary antibody

Reagent	Incubation

mRIPK3 8G7 working 1:500	1 h

Wash

Reagent	Incubation	Cycles
Wash Buffer	2 min	10

Wash

Wash

Wash

Primary antibody

Reagent	Incubation
Flex Peroxidase Blcok working	4 min

Wash

Reagent	Incubation	Cycles
Wash Buffer	2 min	10

Wash

Wash

Wash

Primary antibody

Reagent	Incubation
Protein Block X0909 (Dako) RTU	10 min

Wash

Reagent	Incubation	Cycles
Wash Buffer	2 min	10

Wash

Wash

Wash

Endogenous enzyme block

Wash

Wash

Wash

Wash

Secondary reagent

Wash

Wash

Wash

Wash

Secondary reagent

Wash

Wash

Wash

Wash

Labeled polymer

Reagent	Incubation
HRP (anti-rat)	30 min

Wash

Reagent	Incubation	Cycles
Wash Buffer	2 min	10

Wash

Reagent	Incubation	Cycles
Wash Buffer	2 min	10

Wash

Reagent	Incubation	Cycles
DI Water	31 s	1

Wash

Reagent	Incubation	Cycles
Wash Buffer	2 min	10

Substrate chromogen

Reagent	Incubation
EnV FLEX HRP Magenta Substrate Working Solution	5 min

Wash

Reagent	Incubation	Cycles
Wash Buffer	2 min	10

Substrate chromogen

Wash

Reagent	Incubation	Cycles
Wash Buffer	2 min	10

Wash

Reagent	Incubation	Cycles
DI Water	31 s	1

Wash

Reagent	Incubation	Cycles
Wash Buffer	2 min	10

Endogenous enzyme block

Reagent	Incubation
DA - Sulfuric acid	10 min

Wash

Reagent	Incubation	Cycles
Wash Buffer	2 min	10

Wash

Wash

Wash

Protein block

Wash

Primary antibody

Reagent	Incubation
SMA [D4K9N] #19245 Working 1:300	1 h

Wash

Reagent	Incubation	Cycles
Wash Buffer	2 min	10

Wash

Wash

Wash

Primary antibody

Reagent	Incubation
Flex Peroxidase Blcok	4 min

working

Wash

Reagent	Incubation	Cycles
Wash Buffer	2 min	10

Wash

Wash

Wash

Primary antibody

Reagent	Incubation
Rabbit Labelled Polymer - HRP (Dako K4003)	30 min

Wash

Reagent	Incubation	Cycles
Wash Buffer	2 min	10

Wash

Wash

Wash

Endogenous enzyme block

Wash

Wash

Wash

Wash

Secondary reagent

Wash

Wash

Wash

Wash

Secondary reagent

Wash

Wash

Wash

Wash

Labeled polymer

Reagent	Incubation
Buffer Only	3 min

Wash

Reagent	Incubation	Cycles
Wash Buffer	2 min	10

Wash

Reagent	Incubation	Cycles
Wash Buffer	2 min	10

Wash

Reagent	Incubation	Cycles
DI Water	31 s	1

Wash

Reagent	Incubation	Cycles
Wash Buffer	2 min	10

Substrate chromogen

Reagent	Incubation
EnV FLEX Substrate Working Solution	10 min

Wash

Reagent	Incubation	Cycles
Wash Buffer	2 min	10

Substrate chromogen

Wash

Reagent	Incubation	Cycles
Wash Buffer	2 min	10

Wash

Reagent	Incubation	Cycles
DI Water	31 s	1

Wash

Reagent	Incubation	Cycles
Wash Buffer	2 min	10

Endogenous enzyme block

**Wash**
**Wash**
**Wash**
**Wash**
**Protein block**
**Wash**
**Primary antibody**
**Wash**
**Wash**
**Wash**
**Wash**
**Primary antibody**
**Wash**
**Wash**
**Wash**
**Wash**
**Primary antibody**
**Wash**
**Wash**
**Wash**
**Wash**
**Endogenous enzyme block**
**Wash**
**Wash**
**Wash**
**Wash**
**Secondary reagent**
**Wash**
**Wash**
**Wash**
**Wash**
**Secondary reagent**

**Wash**
**Wash**
**Wash**
**Wash**
**Labeled polymer**
**Wash**
**Wash**
**Wash**
**Wash**
**Substrate chromogen**
**Wash**
**Substrate chromogen**
**Wash**
**Wash**
**Wash**
**Endogenous enzyme block**
**Wash**
**Wash**
**Wash**
**Wash**
**Protein block**
**Wash**
**Primary antibody**
**Wash**
**Wash**
**Wash**
**Wash**
**Primary antibody**
**Wash**
**Wash**
**Wash**
**Wash**

Protocol details

**Primary antibody**
**Wash**
**Wash**
**Wash**
**Wash**
**Endogenous enzyme block**
**Wash**
**Wash**
**Wash**
**Wash**
**Secondary reagent**
**Wash**
**Wash**
**Wash**
**Wash**
**Secondary reagent**
**Wash**
**Wash**
**Wash**
**Wash**
**Labeled polymer**
**Wash**
**Wash**
**Wash**
**Wash**
**Substrate chromogen**
**Wash**
**Substrate chromogen**
**Wash**
**Wash**
**Wash**
**Endogenous enzyme block**

**Wash**
**Wash**
**Wash**
**Wash**
**Protein block**
**Wash**
**Primary antibody**
**Wash**
**Wash**
**Wash**
**Wash**
**Primary antibody**
**Wash**
**Wash**
**Wash**
**Wash**
**Primary antibody**
**Wash**
**Wash**
**Wash**
**Wash**
**Endogenous enzyme block**
**Wash**
**Wash**
**Wash**
**Wash**
**Secondary reagent**
**Wash**
**Wash**
**Wash**
**Wash**
**Secondary reagent**

**Wash**
**Wash**
**Wash**
**Wash**
**Labeled polymer**
**Wash**
**Wash**
**Wash**
**Wash**
**Substrate chromogen**
**Wash**
**Substrate chromogen**
**Wash**
**Wash**
**Wash**
**Endogenous enzyme block**
**Wash**
**Wash**
**Wash**
**Wash**
**Protein block**
**Wash**
**Primary antibody**
**Wash**
**Wash**
**Wash**
**Wash**
**Primary antibody**
**Wash**
**Wash**
**Wash**
**Wash**

Protocol details

**Primary antibody**
**Wash**
**Wash**
**Wash**
**Wash**
**Endogenous enzyme block**
**Wash**
**Wash**
**Wash**
**Wash**
**Secondary reagent**
**Wash**
**Wash**
**Wash**
**Wash**
**Secondary reagent**
**Wash**
**Wash**
**Wash**
**Wash**
**Labeled polymer**
**Wash**
**Wash**
**Wash**
**Wash**
**Substrate chromogen**
**Wash**
**Substrate chromogen**
**Wash**
**Wash**
**Wash**
**Endogenous enzyme block**

Protocol details

**Wash**
**Wash**
**Wash**
**Wash**
**Protein block**
**Wash**
**Primary antibody**
**Wash**
**Wash**
**Wash**
**Wash**
**Primary antibody**
**Wash**
**Wash**
**Wash**
**Wash**
**Primary antibody**
**Wash**
**Wash**
**Wash**
**Wash**
**Endogenous enzyme block**
**Wash**
**Wash**
**Wash**
**Wash**
**Secondary reagent**
**Wash**
**Wash**
**Wash**
**Wash**
**Secondary reagent**

Protocol details

**Wash**
**Wash**
**Wash**
**Wash**
**Labeled polymer**
**Wash**
**Wash**
**Wash**
**Wash**
**Substrate chromogen**
**Wash**
**Substrate chromogen**
**Wash**
**Wash**
**Wash**
**Endogenous enzyme block**
**Wash**
**Wash**
**Wash**
**Wash**
**Protein block**
**Wash**
**Primary antibody**
**Wash**
**Wash**
**Wash**
**Wash**
**Primary antibody**
**Wash**
**Wash**
**Wash**
**Wash**

Protocol details

**Primary antibody**
**Wash**
**Wash**
**Wash**
**Wash**
**Endogenous enzyme block**
**Wash**
**Wash**
**Wash**
**Wash**
**Secondary reagent**
**Wash**
**Wash**
**Wash**
**Wash**
**Secondary reagent**
**Wash**
**Wash**
**Wash**
**Wash**
**Labeled polymer**
**Wash**
**Wash**
**Wash**
**Wash**
**Substrate chromogen**
**Wash**
**Substrate chromogen**
**Wash**
**Wash**
**Wash**
**Endogenous enzyme block**

Protocol details

**Wash**
**Wash**
**Wash**
**Wash**
**Protein block**
**Wash**
**Primary antibody**
**Wash**
**Wash**
**Wash**
**Wash**
**Primary antibody**
**Wash**
**Wash**
**Wash**
**Wash**
**Primary antibody**
**Wash**
**Wash**
**Wash**
**Wash**
**Endogenous enzyme block**
**Wash**
**Wash**
**Wash**
**Wash**
**Secondary reagent**
**Wash**
**Wash**
**Wash**
**Wash**
**Secondary reagent**

**Wash**
**Wash**
**Wash**
**Wash**
**Labeled polymer**
**Wash**
**Wash**
**Wash**
**Wash**
**Substrate chromogen**
**Wash**
**Substrate chromogen**
**Wash**
**Wash**
**Wash**
**Endogenous enzyme block**
**Wash**
**Wash**
**Wash**
**Wash**
**Protein block**
**Wash**
**Primary antibody**
**Wash**
**Wash**
**Wash**
**Wash**
**Primary antibody**
**Wash**
**Wash**
**Wash**
**Wash**

Protocol details

**Primary antibody**
**Wash**
**Wash**
**Wash**
**Wash**
**Endogenous enzyme block**
**Wash**
**Wash**
**Wash**
**Wash**
**Secondary reagent**
**Wash**
**Wash**
**Wash**
**Wash**
**Secondary reagent**
**Wash**
**Wash**
**Wash**
**Wash**
**Labeled polymer**
**Wash**
**Wash**
**Wash**
**Wash**
**Substrate chromogen**
**Wash**
**Substrate chromogen**
**Wash**
**Wash**
**Wash**

Counterstaining

Walter & Eliza Hall Institute of Medical Research
Royal Parade
PARKVILLE VIC 3052

Protocol - hMLKL (Abcam EPR17514)

Version: 8/03/2023 1:00 PM

Dewax

Two phase dewax IHC

Solvent	Transport fluid	Temperature	Incubation top	Incubation bottom	Cycles
Clearify Clearing Agent	DI Water	25 °C	10 s	1 min	1

Two phase dewax wash IHC

Reagent	Incubation	Cycles
DI Water	5 s	1

Target retrieval

Target retrieval IHC

Reagent	Temperature	Incubation	Cooling fluid
EnV FLEX TRS, High pH	97 °C	30 min	DI Water

Non target retrieval wash IHC

Staining

Wash

Reagent	Incubation	Cycles
Wash Buffer	2:40 min	2

Enzyme pre-treatment

Reagent	Incubation
Background Sniper BS966L (Biocare Medical) working	10 min

Wash

Reagent	Incubation	Cycles
Wash Buffer	2 min	10

Wash

Endogenous enzyme block

Wash

Protein block

Wash

Protocol details

Primary antibody

Reagent	Incubation
hMLKL (Abcam EPR17514) working 1:500	1 h

Wash

Reagent	Incubation	Cycles
Wash Buffer	2 min	10

Wash

Wash

Wash

Primary antibody

Reagent	Incubation
Flex Peroxidase Blcok working	4 min

Wash

Reagent	Incubation	Cycles
Wash Buffer	2 min	10

Wash

Wash

Wash

Primary antibody

Reagent	Incubation
MACH4 Rabbit RTU	30 min

Wash

Reagent	Incubation	Cycles
Wash Buffer	2 min	10

Wash

Wash

Wash

Endogenous enzyme block

Wash

Secondary reagent

Wash

Wash

Wash

Wash

Secondary reagent

Wash

Wash

Wash

Wash

Labeled polymer

Reagent	Incubation
Buffer Only	3 min

Wash

Reagent	Incubation	Cycles
Wash Buffer	2 min	10

Wash

Reagent	Incubation	Cycles
Wash Buffer	2 min	10

Wash

Reagent	Incubation	Cycles
DI Water	31 s	1

Wash

Reagent	Incubation	Cycles
Wash Buffer	2 min	10

Wash

Wash

Wash

Substrate chromogen

Reagent	Incubation
EnV FLEX Substrate Working Solution	10 min

Wash

Substrate chromogen

Wash

Reagent	Incubation	Cycles
Wash Buffer	2 min	10

Wash

Reagent	Incubation	Cycles
DI Water	31 s	1

Wash

Reagent	Incubation	Cycles
Wash Buffer	2 min	10

Counterstaining

Walter & Eliza Hall Institute of Medical Research
Royal Parade
PARKVILLE VIC 3052

Agilent Dako

Protocol - hMLKL [10C2] 1:500 LRT

Version: 30/11/2023 3:46 PM

Dewax

Two phase dewax IHC

Solvent	Transport fluid	Temperature	Incubation top	Incubation bottom	Cycles
Clearify Clearing Agent	DI Water	25 °C	10 s	1 min	1

Two phase dewax wash IHC

Reagent	Incubation	Cycles
DI Water	5 s	1

Target retrieval

Target retrieval IHC

Reagent	Temperature	Incubation	Cooling fluid
EnV FLEX TRS, High pH	97 °C	40 min	DI Water

Non target retrieval wash IHC

Staining

Wash

Reagent	Incubation	Cycles
Wash Buffer	2:40 min	2

Enzyme pre-treatment

Wash

Wash

Endogenous enzyme block

Wash

Protein block

Reagent	Incubation
Protein Block RTU X0909 (Dako)	10 min

Wash

Reagent	Incubation	Cycles
Wash Buffer	2 min	10

Primary antibody

**Reagent**	**Incubation**	
hMLKL [10C2] 1:500	1 h	
**Wash**		
**Reagent**	**Incubation**	**Cycles**
Wash Buffer	2 min	10
**Wash**		
**Wash**		
**Wash**		
**Primary antibody**		
**Reagent**	**Incubation**	
Flex Peroxidase Blcok working	4 min	
**Wash**		
**Reagent**	**Incubation**	**Cycles**
Wash Buffer	2 min	10
**Wash**		
**Wash**		
**Wash**		
**Primary antibody**		
**Reagent**	**Incubation**	
Rat HRP (Vector)	30 min	
**Wash**		
**Reagent**	**Incubation**	**Cycles**
Wash Buffer	2 min	10
**Wash**		
**Wash**		
**Wash**		
**Endogenous enzyme block**		
**Wash**		
**Secondary reagent**		
**Reagent**	**Incubation**	
amp reagent A	10 min	
**Wash**		

Reagent	Incubation	Cycles
Wash Buffer	2 min	10

Wash

Reagent	Incubation	Cycles
Wash Buffer	2 min	10

Wash

Wash

Secondary reagent

Wash

Wash

Wash

Wash

Labeled polymer

Reagent	Incubation
Ampb	30 min

Wash

Reagent	Incubation	Cycles
Wash Buffer	2 min	10

Wash

Reagent	Incubation	Cycles
Wash Buffer	2 min	10

Wash

Reagent	Incubation	Cycles
DI Water	31 s	1

Wash

Reagent	Incubation	Cycles
Wash Buffer	2 min	10

Wash

Wash

Wash

Substrate chromogen

Reagent	Incubation
EnV FLEX Substrate	

Working Solution	5 min

Wash

Reagent	Incubation	Cycles
Wash Buffer	2 min	10

Substrate chromogen

Wash

Reagent	Incubation	Cycles
Wash Buffer	2 min	10

Wash

Reagent	Incubation	Cycles
DI Water	31 s	1

Wash

Reagent	Incubation	Cycles
Wash Buffer	2 min	10

Counterstaining

Walter & Eliza Hall Institute of Medical Research
Royal Parade
PARKVILLE VIC 3052

Agilent Dako

Protocol - hRIPK3 [E7A7F] CST10188 IHC-DAB

Version: 5/12/2023 11:59 AM

Dewax

Two phase dewax IHC

Solvent	Transport fluid	Temperature	Incubation top	Incubation bottom	Cycles
Clearify Clearing Agent	DI Water	25 °C	10 s	1 min	1

Two phase dewax wash IHC

Reagent	Incubation	Cycles
DI Water	5 s	1

Target retrieval

Target retrieval IHC

Reagent	Temperature	Incubation	Cooling fluid
EnV FLEX TRS, High pH	97 °C	30 min	DI Water

Non target retrieval wash IHC

Staining

Wash

Reagent	Incubation	Cycles
Wash Buffer	2:40 min	2

Enzyme pre-treatment

Wash

Wash

Endogenous enzyme block

Wash

Protein block

Wash

Primary antibody

Reagent	Incubation
hRIPK3 1:100 [E7A7F] CST10188	1 h

Wash

Reagent	Incubation	Cycles

Wash Buffer	2 min	10

Wash

Wash

Wash

Primary antibody

Reagent	Incubation
Flex Peroxidase Blcok working	4 min

Wash

Reagent	Incubation	Cycles
Wash Buffer	2 min	10

Wash

Wash

Wash

Primary antibody

Reagent	Incubation
Rabbit Labelled Polymer - HRP (Dako K4003)	30 min

Wash

Reagent	Incubation	Cycles
Wash Buffer	2 min	10

Wash

Wash

Wash

Endogenous enzyme block

Wash

Secondary reagent

Wash

Wash

Wash

Wash

Secondary reagent

Wash

Wash

Wash

Wash

Labeled polymer

Reagent	Incubation
Buffer Only	3 min

Wash

Reagent	Incubation	Cycles
Wash Buffer	2 min	10

Wash

Reagent	Incubation	Cycles
Wash Buffer	2 min	10

Wash

Reagent	Incubation	Cycles
DI Water	31 s	1

Wash

Reagent	Incubation	Cycles
Wash Buffer	2 min	10

Wash

Wash

Wash

Substrate chromogen

Reagent	Incubation
EnV FLEX Substrate Working Solution	10 min

Wash

Substrate chromogen

Wash

Reagent	Incubation	Cycles
Wash Buffer	2 min	10

Wash

Reagent	Incubation	Cycles

DI Water	31 s	1

Wash

Reagent	Incubation	Cycles
Wash Buffer	2 min	10

Counterstaining

Walter & Eliza Hall Institute of Medical Research
Royal Parade
PARKVILLE VIC 3052

Protocol - hCaspase-8 (clone B.925.8)

Version: 18/05/2023 3:34 PM

Dewax

Two phase dewax IHC

Solvent	Transport fluid	Temperature	Incubation top	Incubation bottom	Cycles
Clearify Clearing Agent	DI Water	25 °C	10 s	1 min	1

Two phase dewax wash IHC

Reagent	Incubation	Cycles
DI Water	5 s	1

Target retrieval

Target retrieval IHC

Reagent	Temperature	Incubation	Cooling fluid
EnV FLEX TRS, High pH	97 °C	20 min	DI Water

Non target retrieval wash IHC

Staining

Wash

Reagent	Incubation	Cycles
Wash Buffer	2:40 min	2

Enzyme pre-treatment

Reagent	Incubation
Protein Block X0909 (Dako) RTU working	10 min

Wash

Reagent	Incubation	Cycles
Wash Buffer	2 min	10

Wash

Endogenous enzyme block

Wash

Protein block

Wash

Primary antibody

Protocol details

Reagent	Incubation	
hCaspase-8 (clone B.925.8) working 1:50	1 h	
**Wash**		
**Reagent**	**Incubation**	**Cycles**
Wash Buffer	2 min	10
**Wash**		
**Wash**		
**Wash**		
**Primary antibody**		
**Reagent**	**Incubation**	
Flex Peroxidase Blcok working	4 min	
**Wash**		
**Reagent**	**Incubation**	**Cycles**
Wash Buffer	2 min	10
**Wash**		
**Wash**		
**Wash**		
**Primary antibody**		
**Reagent**	**Incubation**	
Mouse HRP Dako RTU	30 min	
**Wash**		
**Reagent**	**Incubation**	**Cycles**
Wash Buffer	2 min	10
**Wash**		
**Wash**		
**Wash**		
**Endogenous enzyme block**		
**Wash**		
**Secondary reagent**		
**Wash**		
**Wash**		

Wash

Wash

Secondary reagent

Wash

Wash

Wash

Wash

Labeled polymer

Reagent	Incubation
Buffer Only	3 min

Wash

Reagent	Incubation	Cycles
Wash Buffer	2 min	10

Wash

Reagent	Incubation	Cycles
Wash Buffer	2 min	10

Wash

Reagent	Incubation	Cycles
DI Water	31 s	1

Wash

Reagent	Incubation	Cycles
Wash Buffer	2 min	10

Wash

Wash

Wash

Substrate chromogen

Reagent	Incubation
EnV FLEX Substrate Working Solution	10 min

Wash

Substrate chromogen

Wash

Reagent	Incubation	Cycles

Wash Buffer	2 min	10

Wash

Reagent	Incubation	Cycles
DI Water	31 s	1

Wash

Reagent	Incubation	Cycles
Wash Buffer	2 min	10

Counterstaining

Walter & Eliza Hall Institute of Medical Research
Royal Parade
PARKVILLE VIC 3052

Agilent Dako

Protocol - c-caspase 3 MACH4 CST 9661 (Ap 175)

Version: 26/07/2023 8:59 AM

Dewax

Two phase dewax IHC

Solvent	Transport fluid	Temperature	Incubation top	Incubation bottom	Cycles
Clearify Clearing Agent	DI Water	25 °C	10 s	1 min	1

Two phase dewax wash IHC

Reagent	Incubation	Cycles
DI Water	5 s	1

Target retrieval

Target retrieval IHC

Reagent	Temperature	Incubation	Cooling fluid
EnV FLEX TRS, High pH	97 °C	40 min	DI Water

Non target retrieval wash IHC

Staining

Wash

Reagent	Incubation	Cycles
Wash Buffer	2:40 min	2

Enzyme pre-treatment

Wash

Wash

Endogenous enzyme block

Wash

Protein block

Wash

Primary antibody

Reagent	Incubation
cleaved caspase 3 working 1:300 CSC	1 h

Wash

Reagent	Incubation	Cycles

Wash Buffer	2 min	10

Wash

Wash

Wash

Primary antibody

Reagent	Incubation
Flex Peroxidase Blcok working	4 min

Wash

Reagent	Incubation	Cycles
Wash Buffer	2 min	10

Wash

Wash

Wash

Primary antibody

Reagent	Incubation
MACH4 Rabbit RTU	30 min

Wash

Reagent	Incubation	Cycles
Wash Buffer	2 min	10

Wash

Wash

Wash

Endogenous enzyme block

Wash

Secondary reagent

Wash

Wash

Wash

Wash

Secondary reagent

Wash

Wash

Wash

Wash

Labeled polymer

Reagent	Incubation
Buffer Only	3 min

Wash

Reagent	Incubation	Cycles
Wash Buffer	2 min	10

Wash

Reagent	Incubation	Cycles
Wash Buffer	2 min	10

Wash

Reagent	Incubation	Cycles
DI Water	31 s	1

Wash

Reagent	Incubation	Cycles
Wash Buffer	2 min	10

Wash

Wash

Wash

Substrate chromogen

Reagent	Incubation
EnV FLEX Substrate Working Solution	10 min

Wash

Substrate chromogen

Wash

Reagent	Incubation	Cycles
Wash Buffer	2 min	10

Wash

Reagent	Incubation	Cycles
DI Water	31 s	1

Wash

Reagent	Incubation	Cycles
Wash Buffer	2 min	10

Counterstaining

Protocol - Ki67 RbxMo 1:400 (H) CSC#12202 RUO

Version: 14/09/2023 9:48 AM

Dewax

Two phase dewax IHC

Solvent	Transport fluid	Temperature	Incubation top	Incubation bottom	Cycles
Clearify Clearing Agent	DI Water	25 °C	10 s	1 min	1

Two phase dewax wash IHC

Reagent	Incubation	Cycles
DI Water	5 s	1

Target retrieval

Target retrieval IHC

Reagent	Temperature	Incubation	Cooling fluid
EnVision FLEX TRS, Low pH	97 °C	30 min	DI Water

Non target retrieval wash IHC

Staining

Wash

Reagent	Incubation	Cycles
Wash Buffer	2:40 min	2

Enzyme pre-treatment

Wash

Wash

Endogenous enzyme block

Wash

Protein block

Wash

Primary antibody

Reagent	Incubation
Ki67 RbxMo 1:400 (L) (D3B5) CSC#12202S working	1 h

Wash

Protocol details

Reagent	Incubation	Cycles
Wash Buffer	2 min	10

Wash

Wash

Wash

Primary antibody

Reagent	Incubation
Flex Peroxidase Blcok working	4 min

Wash

Reagent	Incubation	Cycles
Wash Buffer	2 min	10

Wash

Wash

Wash

Primary antibody

Reagent	Incubation
Rabbit Labelled Polymer - HRP (Dako K4003)	30 min

Wash

Reagent	Incubation	Cycles
Wash Buffer	2 min	10

Wash

Wash

Wash

Endogenous enzyme block

Wash

Secondary reagent

Wash

Wash

Wash

Wash

Secondary reagent

Walter & Eliza Hall Institute of Medical Research
Royal Parade
PARKVILLE VIC 3052

Wash

Wash

Wash

Wash

Labeled polymer

Reagent	Incubation
Buffer Only	3 min

Wash

Reagent	Incubation	Cycles
Wash Buffer	2 min	10

Wash

Reagent	Incubation	Cycles
Wash Buffer	2 min	10

Wash

Reagent	Incubation	Cycles
DI Water	31 s	1

Wash

Reagent	Incubation	Cycles
Wash Buffer	2 min	10

Wash

Wash

Wash

Substrate chromogen

Reagent	Incubation
EnV FLEX Substrate Working Solution	10 min

Wash

Substrate chromogen

Wash

Reagent	Incubation	Cycles
Wash Buffer	2 min	10

Wash

Reagent	Incubation	Cycles

DI Water	31 s	1

Wash

Reagent	Incubation	Cycles
Wash Buffer	2 min	10

Counterstaining
