# Supplementary figures and images for: An immunohistochemical atlas of necroptotic pathway expression
Source: EMBO Mol Med. 2024 May 15;16(7):13. doi: 10.1038/s44321-024-00074-6 (PMC11250867; doi:10.1038/s44321-024-00074-6)

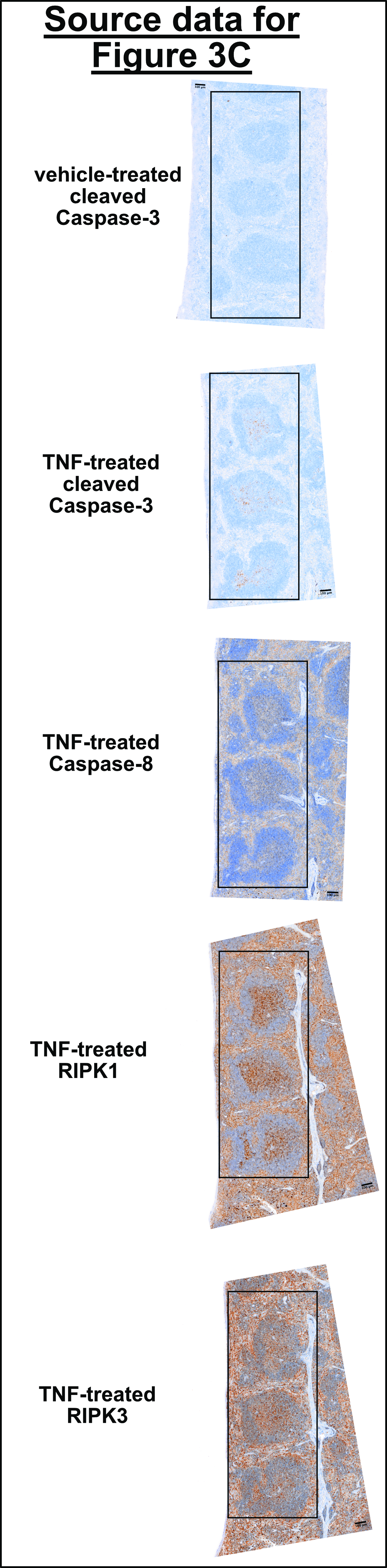

Supplement: Supplementary file 4 — Source data Fig. 3 [file 44321_2024_74_MOESM4_ESM.zip › 240418_source_data_Figure_3C.jpg]

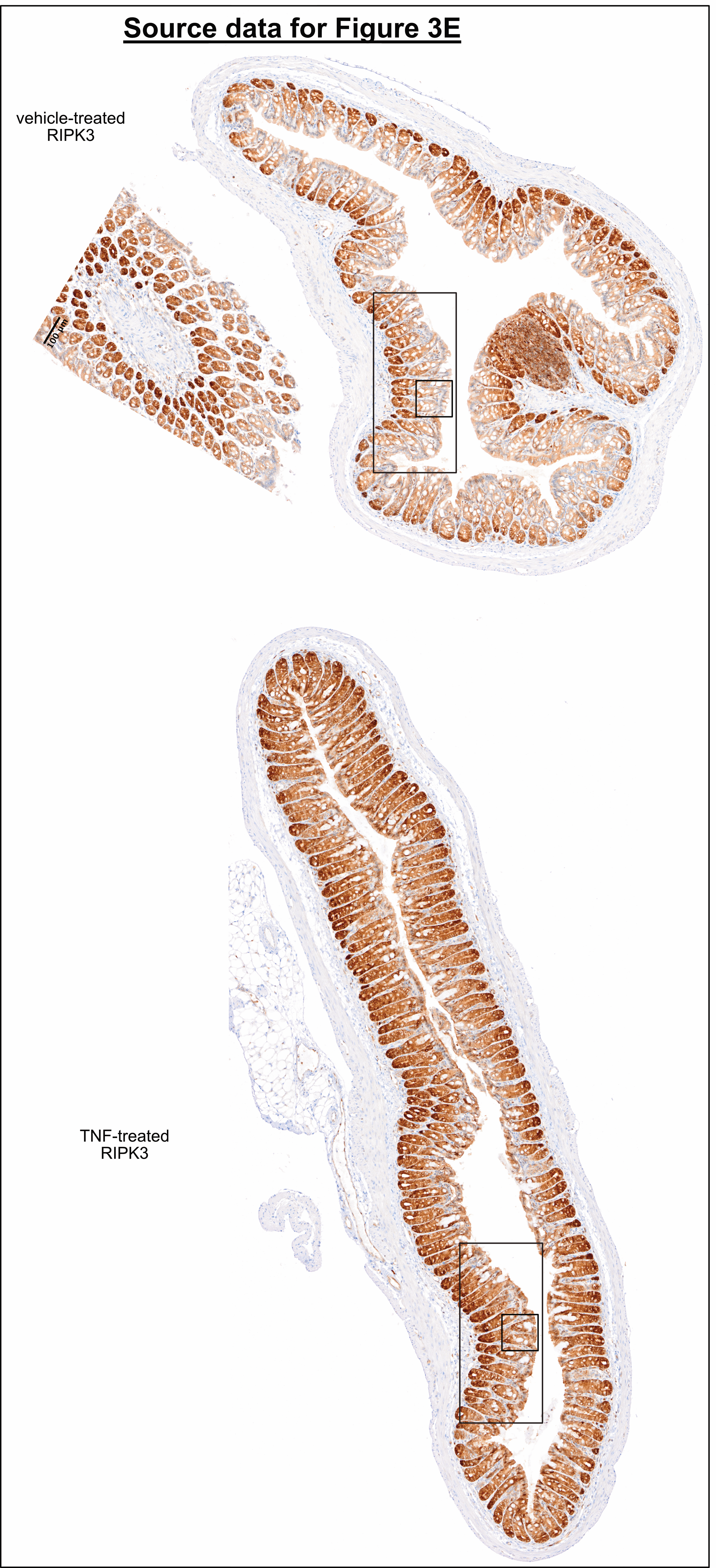

Supplement: Supplementary file 4 — Source data Fig. 3 [file 44321_2024_74_MOESM4_ESM.zip › 240418_source_data_Figure_3E.jpg]

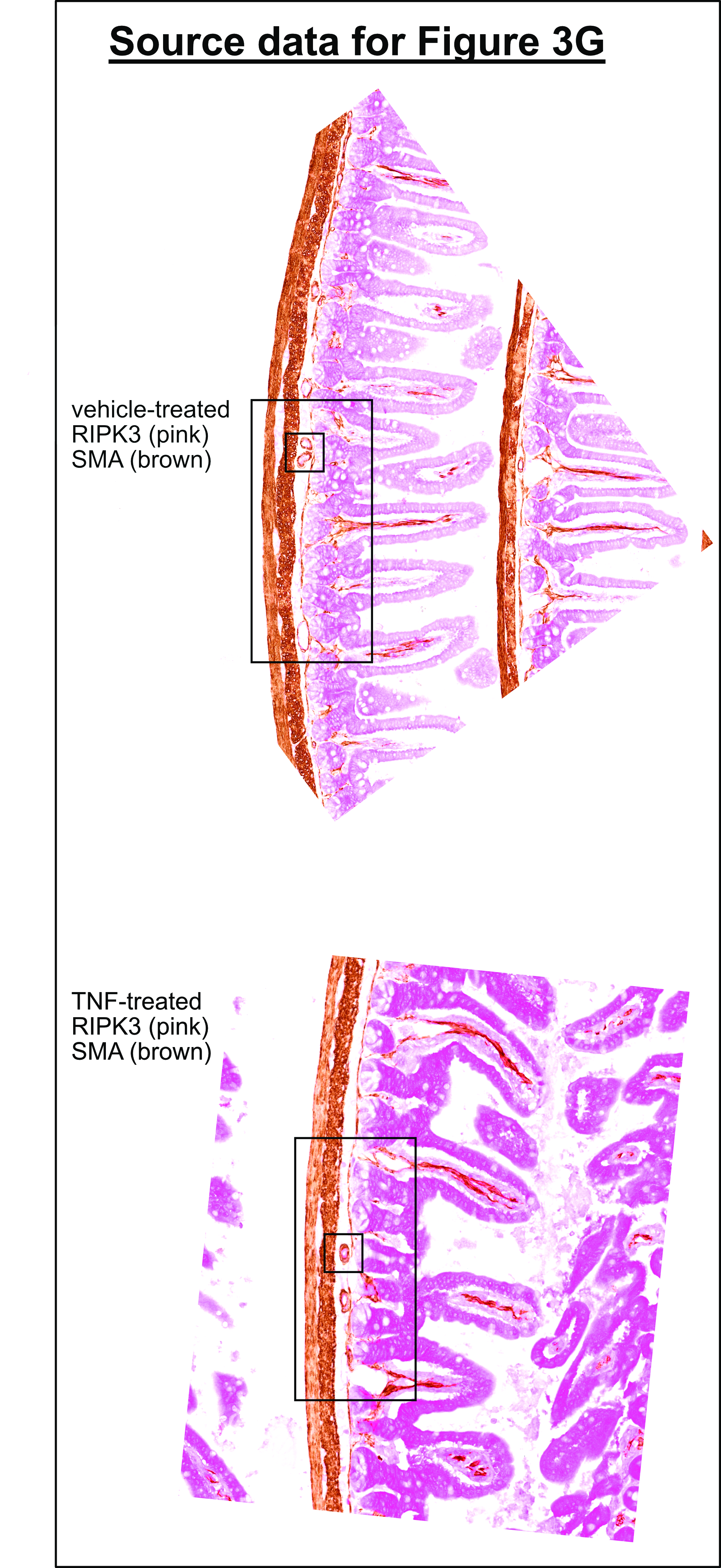

Supplement: Supplementary file 4 — Source data Fig. 3 [file 44321_2024_74_MOESM4_ESM.zip › 240418_source_data_Figure_3G.jpg]

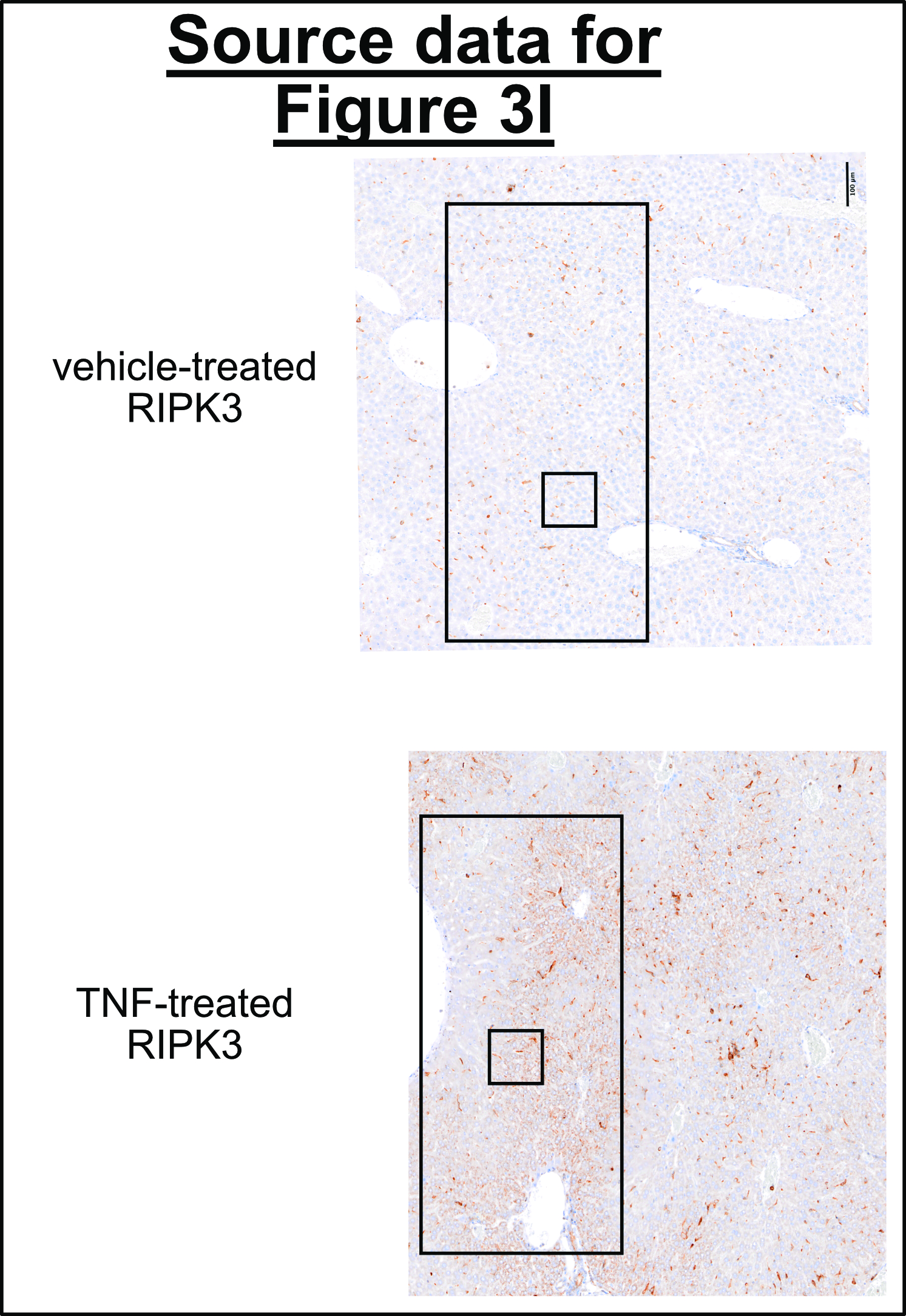

Supplement: Supplementary file 4 — Source data Fig. 3 [file 44321_2024_74_MOESM4_ESM.zip › 240418_source_data_Figure_3I.jpg]

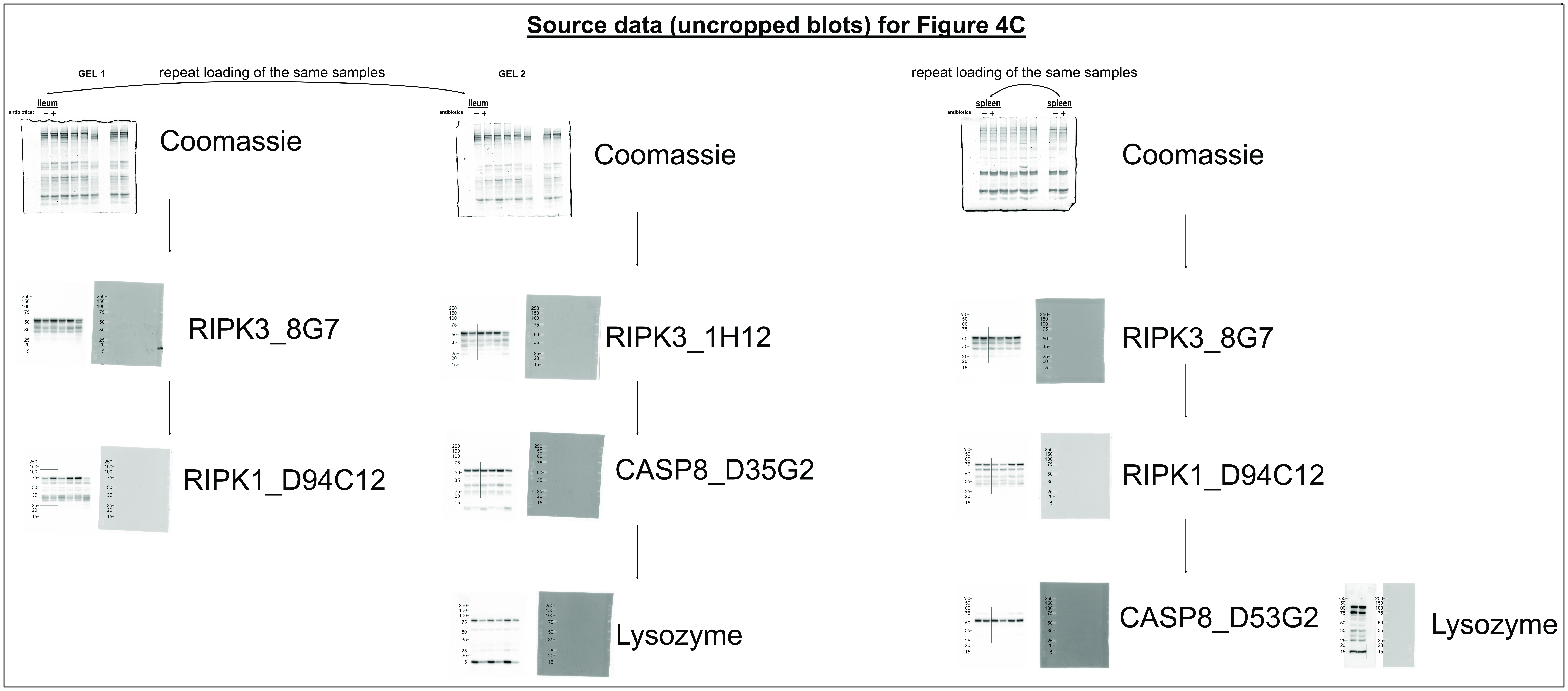

Supplement: Supplementary file 5 — Source data Fig. 4 [file 44321_2024_74_MOESM5_ESM.zip › 240418_source_data_Figure_4C.jpg]

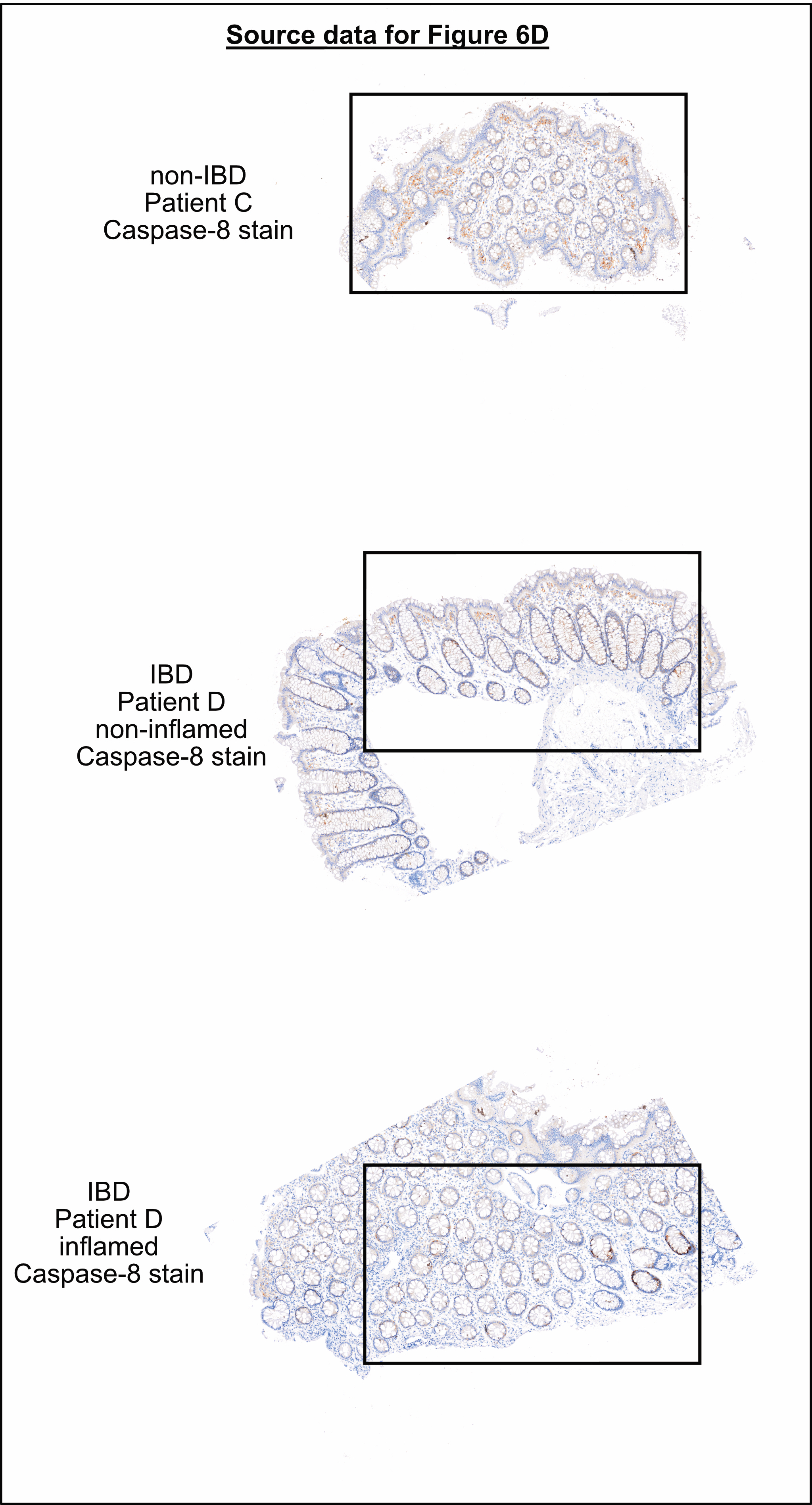

Supplement: Supplementary file 7 — Source data Fig. 6 [file 44321_2024_74_MOESM7_ESM.zip › 240418_source_data_Figure_6D.jpg]
