# Supplementary material for: An immunohistochemical atlas of necroptotic pathway expression
Source: EMBO Mol Med. 2024 May 15;16(7):13. doi: 10.1038/s44321-024-00074-6 (PMC11250867; doi:10.1038/s44321-024-00074-6)
Supplement: Supplementary file 9 — Expanded View Figures [file 44321_2024_74_MOESM9_ESM.pdf]

Expanded View Figures

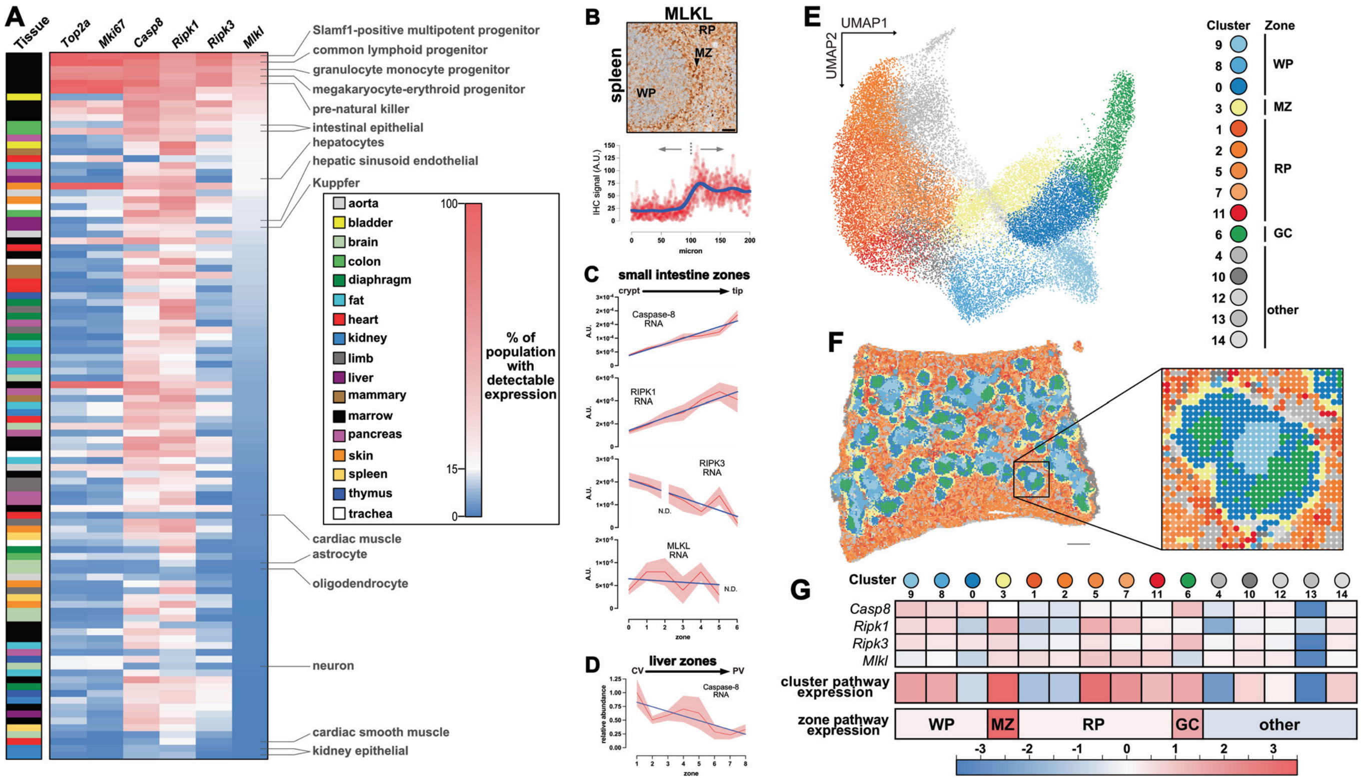

Figure EV1. Constitutive co-expression of necroptotic effectors is confined to fast-cycling cells within progenitors, immune and barrier populations.

(**A**) Heatmap of cell ontologies from the *Tabula Muris* dataset (Tabula Muris et al, 2018). Left-most column depicts the tissue origin of each cell ontology. Other columns indicate the percent of cells within each ontology that expressed *Top2a, Mki67, Casp8, Ripk1, Ripk3*, or *Mlkl*. Legend shows the color-to-tissue and the color-to-frequency scales. Cell ontologies of interest are annotated. (**B**) Micrograph of MLKL immunosignals from the wild-type mouse spleen. The white pulp (WP), marginal zone (MZ), and red pulp (RP) are annotated. Scale bar is 50 μm. Scatterplot shows relative expression levels of MLKL along the white pulp-to-red pulp axis. Red datapoints show immunosignal intensities and the overlaid dark blue line indicates the LOWESS best-fit along $N = 20$ axes from $n = 1$ mouse. Dashed line indicates the boundary between splenic white pulp and marginal zone. Data were representative of $n > 3$ mice. (**C**, **D**) Spatial transcriptomic data from (Moor et al, 2018)) and (Ben-Moshe et al, 2019) showing the relative expression levels (arbitrary units; A.U.) of Caspase-8, RIPK1, RIPK3, or MLKL along the ileal crypt-to-villus axis (**C**) or the hepatic central vein-to-portal vein axis (**D**). (**E**–**G**) Spatial transcriptomic data on mouse spleen 12 days after *Plasmodium berghei*-infection. Panel (**E**) shows a uniform manifold approximation and projection (UMAP) of cell populations distinguished by unsupervised leiden clustering. Legend shows the color assigned to each population. Panel (**F**) shows the location of each cell cluster. Scale bar is 500 μm. Panel (**G**) shows the normalized expression for each gene product. Expression values for *Casp8*, *Ripk1*, *Ripk3*, and *Mlkl* were summated to provide an index of "cluster pathway expression", which was averaged to provide an index of "zone pathway expression". Data were from $n = 1$ mouse.

 © The Author(s)

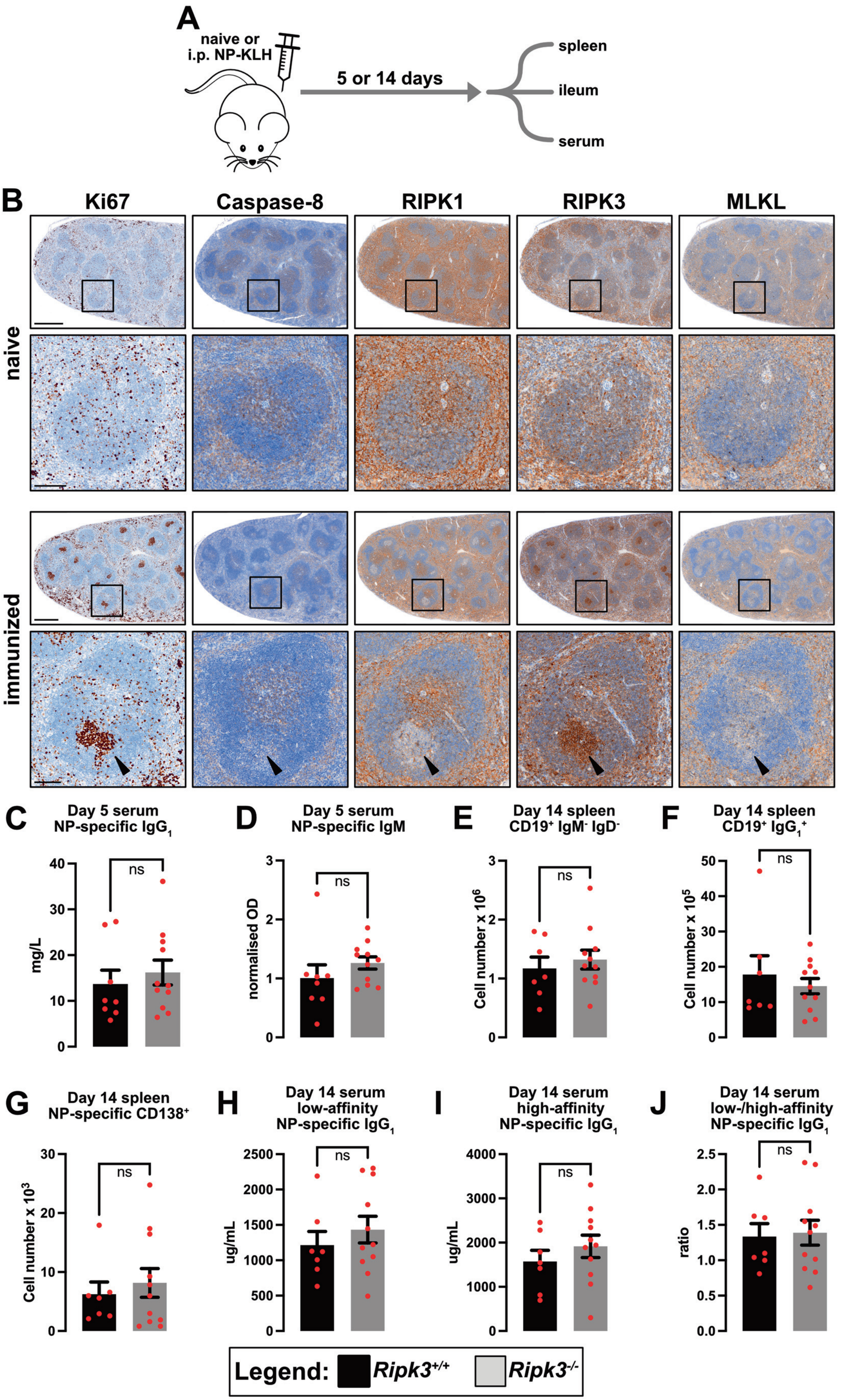
A
naive or
i.p. NP-KLH
5 or 14 days
spleen
ileum
serum
B
Ki67
Caspase-8
RIPK1
RIPK3
MLKL
naive
immunized
C
Day 5 serum
NP-specific IgG1
mg/L
ns
D
Day 5 serum
NP-specific IgM
normalised OD
ns
E
Day 14 spleen
CD19+ IgM- IgD-
Cell number x 10^6
ns
F
Day 14 spleen
CD19+ IgG1+
Cell number x 10^5
ns
G
Day 14 spleen
NP-specific CD138+
Cell number x 10^3
ns
H
Day 14 serum
low-affinity
NP-specific IgG1
ug/mL
ns
I
Day 14 serum
high-affinity
NP-specific IgG1
ug/mL
ns
J
Day 14 serum
low-/high-affinity
NP-specific IgG1
ratio
ns
Legend: Ripk3+/+ Ripk3-/-

Figure EV2. RIPK3 is uniquely upregulated in splenic germinal centers.

(**A**) Experimental design. (**B**) Ki67, Caspase-8, RIPK1, RIPK3, and MLKL immunosignals from adjacent sections of the naïve or NP-KLH-immunized mouse spleen. Arrowheads show a Ki67^{+} germinal center that co-stains for RIPK3, but not other members of the pathway. Representative of *n* > 3 mice per group. Scale bars in lower magnification micrographs are 500 μm. Scale bars in insets are 100 μm. Data were representative of *n* > 3 mice per group. (**C–J**) *Ripk3*$^{-/-}$ or *Ripk3*$^{+/+}$ mice were immunized with NP-KLH and circulating NP-specific IgG_1 (**C**), circulating NP-specific IgM (**D**), splenic mature B cells (**E**, **F**), splenic NP-specific plasma cells (**G**), circulating low affinity NP-specific IgG_1 antibody (**H**), circulating high affinity NP-specific IgG_1 (**I**), and the ratio between circulating low-and-high affinity NP-specific antibody (**J**) were measured at the indicated day after immunization. Bars on graphs in (**C–J**) represent mean ± SEM. Each datapoint represents one mouse. ns non-significant by two-sided *t*-test with Welch's correction.

 © The Author(s)

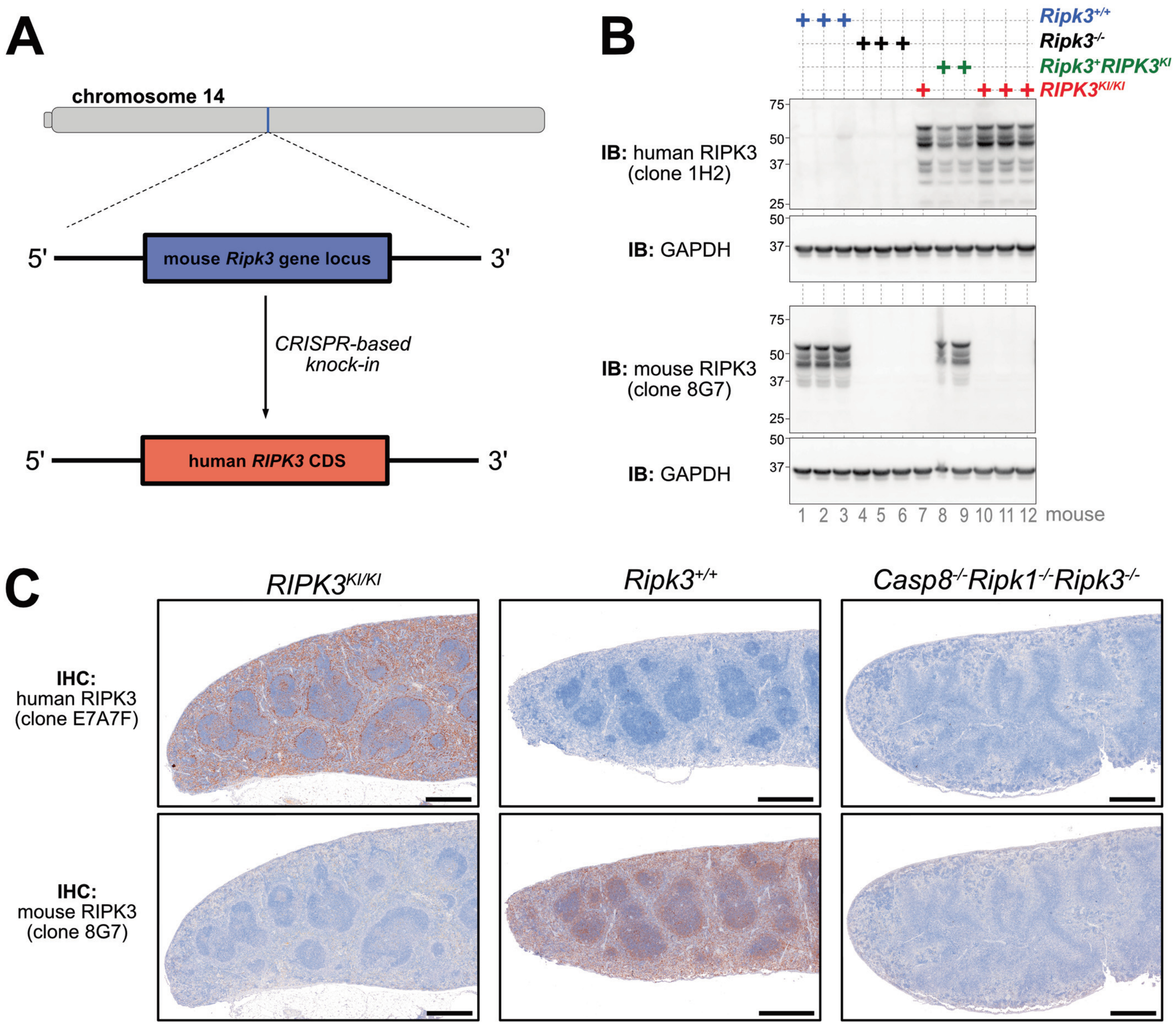

Figure EV3. Assessing the specificity of immunostaining using wild-type, knockout and human RIPK3 knock-in mice.

(**A**) Approach used to insert the human RIPK3 coding sequence (CDS) into the mouse *Ripk3* locus. (**B**) Immunoblot of spleen homogenates from *Ripk3*$^{+/+}$, *Ripk3*$^{-/-}$, human RIPK3 (*RIPK3*$^{KI/KI}$) or hemizygous human RIPK3 (*Ripk3*$^{+}$*RIPK3*^{KI}) mice. GAPDH immunoblots are shown as loading controls. Each lane represents a different mouse. (**C**) Immunosignals produced by the anti-human RIPK3 (clone 37A7F) or anti-mouse RIPK3 (clone 8G7) antibodies on spleen sections from *RIPK3*$^{KI/KI}$, *Ripk3*$^{+/+}$ or *Casp8*$^{-/-}$*Ripk1*$^{-/-}$*Ripk3*$^{-/-}$. Data were representative of $n \geq 3$ for each target and tissue. Scale bars are 500 μm.

© The Author(s)

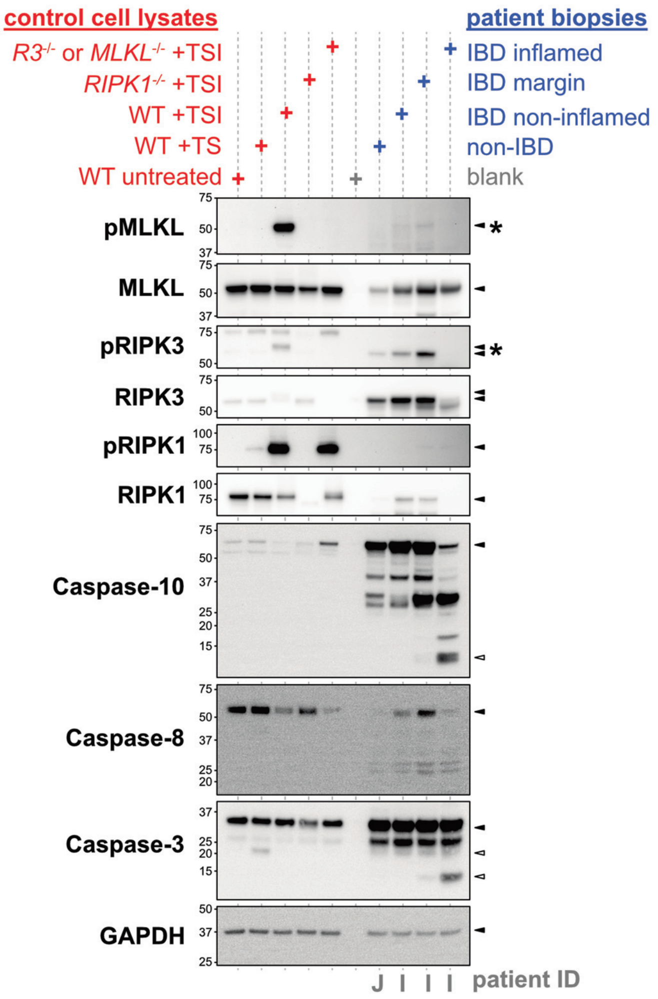

Figure EV4. Another instance of elevated intestinal apoptosis in a patient with ulcerative colitis.

Immunoblot of lysates from HT29 cells (red annotations) and intestinal biopsies from patients A-H (blue annotations). The fifth lane of each gel contained lysates from TSI-treated *RIPK3*$^{-/-}$ or TSI-treated *MLKL*$^{-/-}$ cells (see source data for details). Patient J was a non-IBD control. Patient I had ulcerative colitis (UC). The endoscopic grading of the biopsy site as "non-inflamed", "marginally inflamed", or "inflamed" is stipulated. Closed arrowheads indicate full-length form of proteins. Asterisks indicate active, phosphorylated forms of RIPK3 (pRIPK3) and MLKL (pMLKL). Open arrowheads indicate active, cleaved forms of Caspase-10 and Caspase-3. GAPDH was used as a loading control.

 © The Author(s)

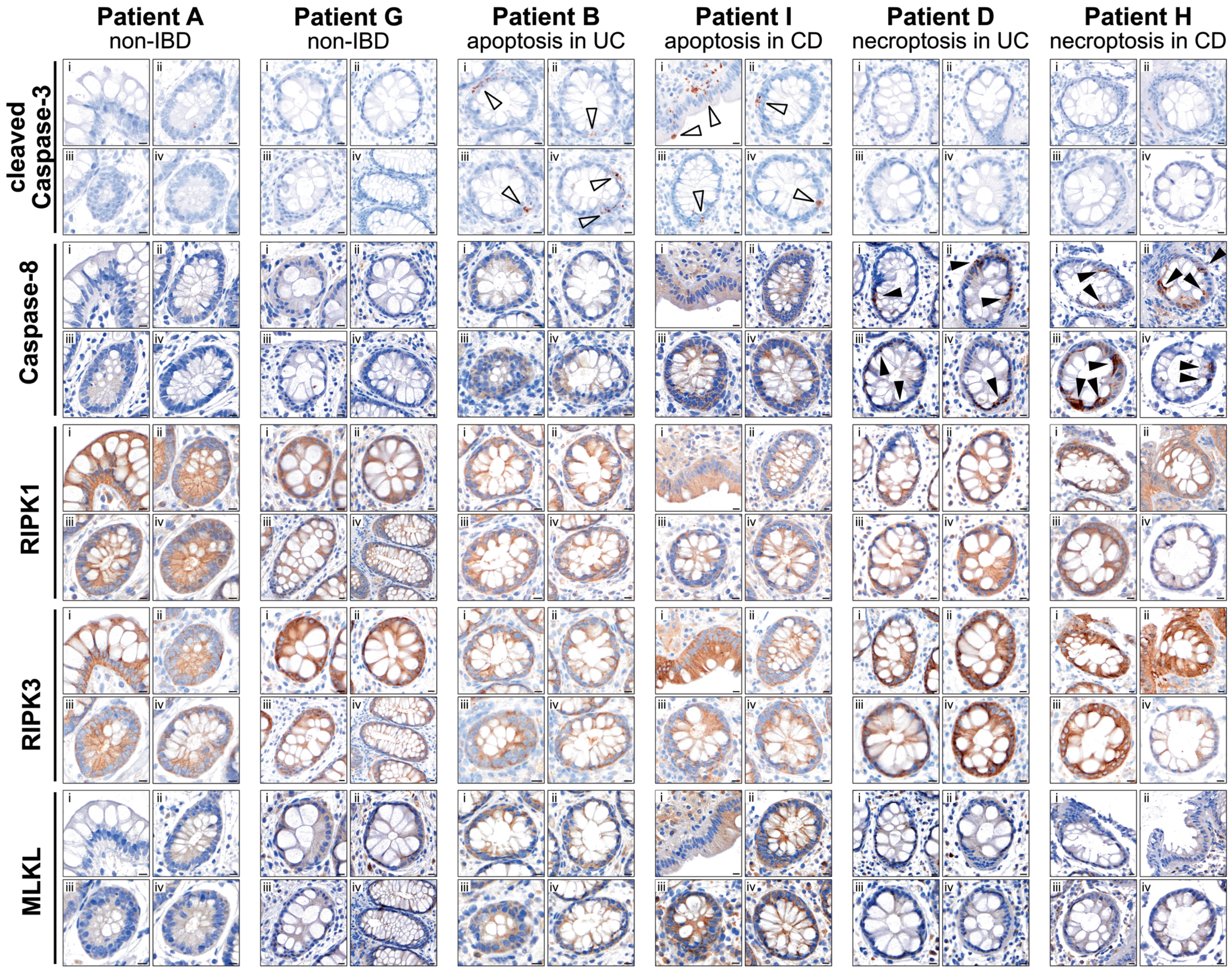

Figure EV5. Atlas of necroptotic pathway expression in human intestinal crypts.

Immunohistochemistry for cleaved Caspase-3, Caspase-8 (clone B.925.8), RIPK1, RIPK3, and MLKL (clone EPR171514) on intestinal biopsies from the stipulated patients. Four representative micrographs per biopsy are shown (i-v). Open arrowheads indicate instances of epithelial apoptosis. Closed arrowheads indicate instances of epithelial Caspase-8 clustering. Scale bars are 10 μm. The location for each micrograph within the biopsy is indicated in Appendix Fig. S5.
